# Bioorthogonal Tools for Ethanolamine Lipids and Protein Conjugates

**DOI:** 10.1002/anie.1866870

**Published:** 2026-08-06

**Authors:** Yuan‐Ting Cho, Cindy Y. Jao, Zijun Xia, Brittany M. White‐Mathieu, Adrian Salic, Jeremy M. Baskin

**Affiliations:** ^1^ Weill Institute for Cell and Molecular Biology Cornell University Ithaca New York USA; ^2^ Department of Chemistry and Chemical Biology Cornell University Ithaca New York USA; ^3^ Department of Cell Biology Harvard Medical School Boston Massachusetts USA; ^4^ Department of Chemistry University of New Hampshire Durham New Hampshire USA

**Keywords:** bioorthogonal chemistry, click chemistry, imaging, lipid metabolism, lipidation, phospholipids

## Abstract

Phosphatidylethanolamine (PE) is the second most abundant class of phospholipids in eukaryotic membranes, as well as a precursor for essential posttranslational protein modifications, such as PE conjugates of ubiquitin and ATG8/LC3 that play key roles in autophagy, and glycosylphosphatidylinositol (GPI) anchors of numerous cell surface proteins. Bioorthogonal chemistry has revolutionized how phospholipid biosynthesis, transport, and turnover are studied, with clickable metabolic precursors now available for several phospholipid classes. Yet no metabolic bioorthogonal probe for labeling endogenous PE and PE‐derived protein modifications has been developed. Here, we introduce an alkyne‐tagged ethanolamine analog (AlkEA) that is incorporated into PE via the Kennedy pathway and can be derivatized by copper‐catalyzed azide–alkyne cycloaddition (CuAAC) for visualization and affinity enrichment. Confocal microscopy revealed the subcellular distribution of AlkEA‐labeled PE in the ER, Golgi, mitochondria, and autophagosomes, while lipidomic analysis demonstrated AlkEA incorporation across diverse PE species. AlkEA labeling also allowed affinity isolation of PE‐conjugated LC3 and ubiquitin, as well as that of a prototypical GPI‐anchored protein. AlkEA is thus a minimally perturbing tool broadly applicable to dissecting PE metabolism and PE‐dependent protein modifications.

## Graphical abstract

An alkyne‐tagged ethanolamine analog (AlkEA) enables bioorthogonal, metabolic labeling of endogenous phosphatidylethanolamine (PE) and its protein modifications. AlkEA is incorporated into PE via the Kennedy pathway, allowing CuAAC‐based visualization of PE and affinity capture of PE‐conjugated LC3, ubiquitin, and GPI‐anchored proteins.

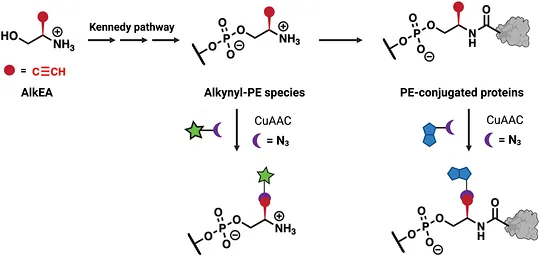

## 1 Introduction

Phosphatidylethanolamine (PE) is the second most abundant glycerophospholipid class in eukaryotes after phosphatidylcholine (PC) [1]. In addition to a structural role in membranes, PE plays diverse functional roles, including as precursor for other lipids [2], regulating membrane curvature and topology [3], supporting mitochondrial biogenesis [4] and oxidative phosphorylation [5], and in the initiation of autophagy [6]. Beyond these roles, PE is also an important precursor to key posttranslational protein modifications. PE supplies phosphoethanolamine groups for the biosynthesis of glycosylphosphatidylinositol (GPI) anchors that tether dozens of proteins to the outer leaflet of the plasma membrane [7, 8, 9]. Additionally, PE is conjugated to the C‐terminus of ubiquitin [10] and ubiquitin‐like proteins [11, 12, 13], most famously those of the ATG8/LC3/GABARAP family, thus playing an essential role in autophagy and the emerging conjugation of ATG8 to single membranes pathway [14, 15]. Despite these myriad functions, the spatiotemporal dynamics of PE metabolism, transport, and posttranslational modifications remain incompletely understood, owing in part to a lack of methods for probing PE and PE‐dependent processes.

Bioorthogonal metabolic labeling is a powerful strategy for interrogating lipid metabolism. This approach relies on the metabolic incorporation of small clickable handles, most commonly terminal alkynes or azides, into native biosynthetic pathways without perturbation of cellular functions [16, 17, 18]. For phospholipids, metabolic labeling was first demonstrated for PC using propargylcholine, an alkyne‐tagged choline analog that is incorporated into all choline‐containing phospholipids [19, 20]. This approach was subsequently extended to other phospholipid classes, including phosphatidylserine (PS) [21], phosphatidylinositol (PI) [22], general glycerophospholipids [23, 24], and phosphatidic acid (PA) synthesis via the phospholipase D pathway [25]. These tools have been used for various applications, including visualization of subcellular sites of lipid synthesis and elucidation of novel regulators of lipid biosynthesis and transport [26, 27, 28, 29, 30, 31, 32, 33]. However, metabolic probes for labeling PE have thus far not been described.

Here, we report the synthesis and characterization of an alkynyl ethanolamine analog ((*R*)‐AlkEA, or AlkEA) that is incorporated into PE, with the terminal alkyne moiety enabling downstream functionalization via CuAAC. We demonstrate that AlkEA recapitulates the subcellular distribution of PE in membranes and allows labeling of proteins bearing PE‐derived posttranslational modifications, including PE conjugates of LC3 and ubiquitin, and GPI anchors. This work provides a physiological and versatile bioorthogonal tool for dissecting the metabolism and diverse biological roles of PE.

## 2 Results and Discussion

### 2.1 Design and Synthesis of Alkynyl Ethanolamine Analogs for PE Metabolic Labeling

Our PE labeling strategy relies on the Kennedy pathway, wherein ethanolamine is converted to phosphoethanolamine and then CDP‐ethanolamine, followed by coupling to diacylglycerol (DAG) to yield PE (Figure 1A,B). The resulting alkyne‐labeled PE species could then be functionalized via CuAAC with an azido fluorophore or biotin derivative, for imaging or biochemical analysis.

**FIGURE 1:**
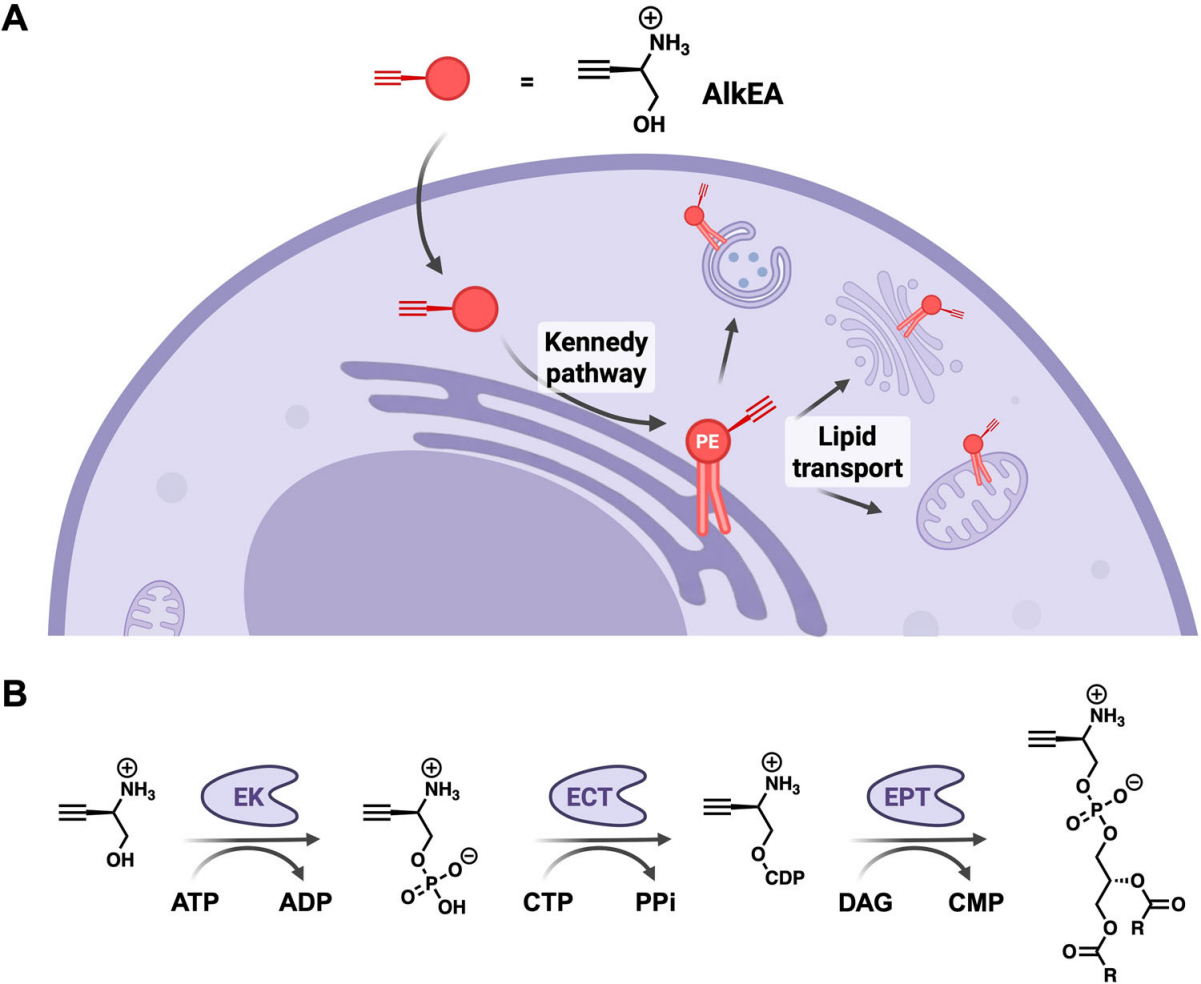
Metabolic labeling of PE using an alkynyl analog of ethanolamine (AlkEA). (A) Schematic of AlkEA utilization by Kennedy pathway enzymes, resulting in the synthesis of alkyne‐labeled PE in the ER, followed by transport to other cellular organelles. (B) Kennedy pathway reactions for AlkEA. R = fatty acyl chains. EK: ethanolamine kinase; ECT: phosphoethanolamine cytidylyltransferase; EPT: ethanolaminephosphotransferase.

Classic studies demonstrated that various aminoalcohols can be incorporated into phospholipids in place of ethanolamine in houseflies [34, 35]; among efficiently incorporated compounds are ethanolamines substituted in the 2 position (2‐aminopropan‐1‐ol, 2‐aminobutan‐1‐ol) or 1 position (1‐aminopropan‐2‐ol). We thus hypothesized that analogs bearing an ethynyl group attached to one of the two ethanolamine carbons might be good metabolic labels for PE. Indeed, 2‐substituted ethynyl analogs, hereby referred to as AlkEA, showed good incorporation and were further characterized (vide infra).

Because installation of the alkyne moiety generates a stereogenic center, both enantiomers of AlkEA were synthesized in enantioenriched form (77%–79% ee, see Synthetic Methods, Supporting Information). The synthesis began with enantiomeric commercially available oxazolidine diester enantiomers **1** and **4** (Scheme 1). The methyl ester was reduced to the corresponding aldehyde using diisobutylaluminum hydride, followed by reaction with the Bestmann–Ohira reagent to afford the desired alkynes **3** and **6**. Subsequent acid‐mediated deprotection yielded the final compounds, (*R*)‐ and (*S*)‐AlkEA.

**SCHEME 1:**
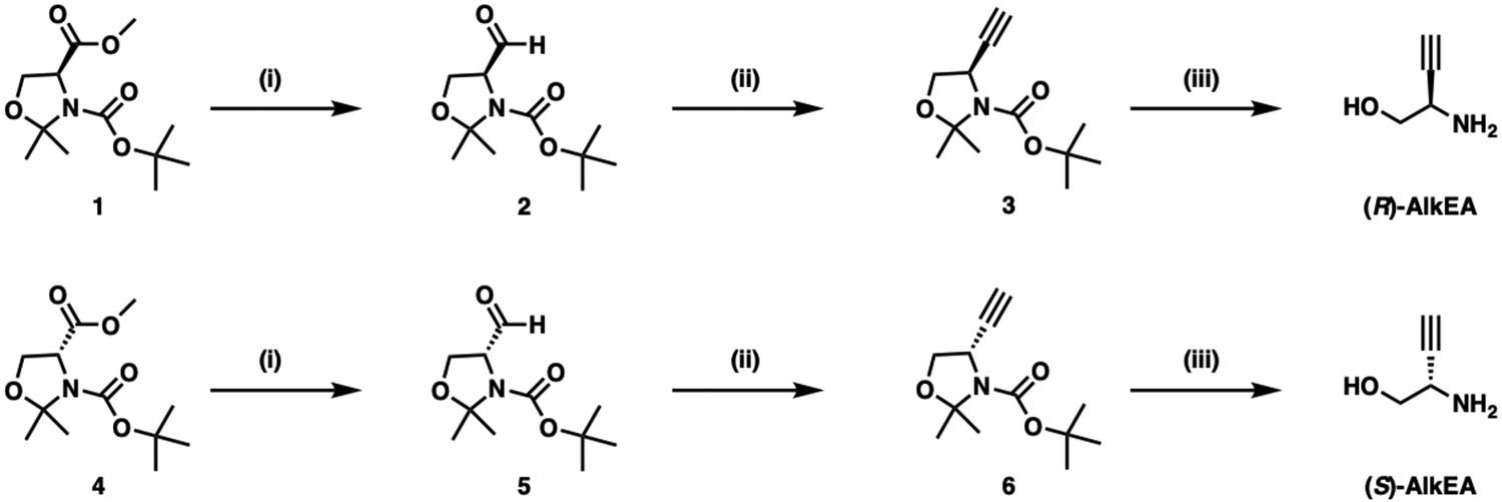
Synthesis of (*R*)‐ and (*S*)‐AlkEA. (i) DIBAL, CH_2_Cl_2_, −80°C, 1 h. (ii) Bestmann–Ohira reagent, K_2_CO_3_, MeOH, 0°C to rt, 3 h, 44% for **3** (two steps from **1**), 41% for **6** (two steps from **4**). (iii) AcCl, MeOH, 0°C to rt, 12 h, 99% for (*R*)‐AlkEA, 95% for (*S*)‐AlkEA.

### 2.2 Imaging Subcellular Distribution of AlkEA‐Labeled PE

We first assessed whether our enantiomeric ethanolamine probes could metabolically label PE in cultured mammalian cells. HeLa cells were incubated with (*R*)‐AlkEA or (*S*)‐AlkEA for 24 h, followed by fixation and CuAAC reaction with Alexa Fluor 488‐azide (Figure 2A). By confocal microscopy, specific fluorescent staining was observed across multiple organelles, consistent with the widespread subcellular distribution of PE (Figure 2B). Notably, the (*R*)‐AlkEA signal was stronger and more obviously localized to membranes, whereas the (*S*)‐AlkEA signal was weaker and more diffuse, possibly due to lower utilization by the Kennedy pathway and/or off‐target labeling (Figure S1). Based on these results, we used (*R*)‐AlkEA (and refer to it herein as AlkEA) in all subsequent experiments. We used the MTT assay to assess cellular toxicity and found that AlkEA concentrations of at least 10 mM caused no reduction in cell viability (Figure S2).

**FIGURE 2:**
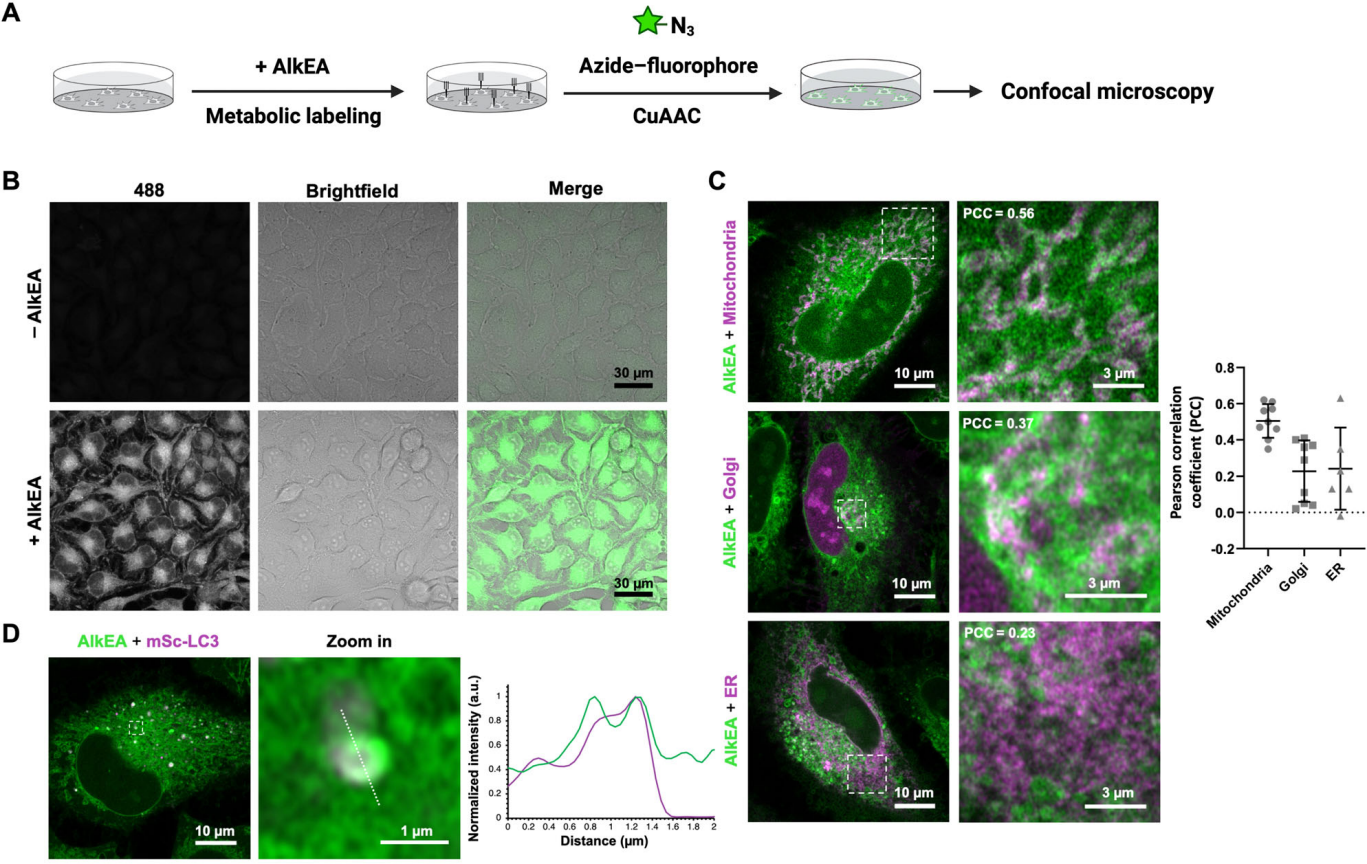
Imaging of AlkEA‐labeled PE by fluorescence microscopy. (A) Schematic of microscopy workflow. HeLa cells are incubated with AlkEA (1 mM, 24 h) or vehicle, followed by CuAAC reaction with Alexa Fluor 488‐azide and confocal imaging. (B) AlkEA‐labeled lipids localize to cellular membranes. (C) As in (B), but showing colocalization of AlkEA‐labeled lipids (green) with fluorescent organelle markers (magenta). Organelle markers: ER (Sec61β‐mRFP), Golgi (mCherry‐PH(OSBP)), and mitochondria (OMP25TM‐mCherry). Pearson correlation coefficients (PCC) are shown in the right panels; bars indicate mean ± SD. (D) AlkEA punctate signal (green) colocalizes with mScarlet‐i‐LC3 (magenta) at autophagosomal membranes. The inset shows a magnified view of the boxed region. The right panel shows fluorescence intensity along the indicated line, confirming spatial overlap between AlkEA‐labeled lipids and LC3‐positive puncta. a.u.: arbitrary units.

We determined the subcellular localization of AlkEA‐labeled PE using cells expressing fluorescent protein markers for several organelles. Significant overlap was observed between AlkEA‐labeled PE and the endoplasmic reticulum (ER), mitochondria, and Golgi apparatus (Figure 2C). This organelle distribution is consistent with known PE metabolism, as PE is synthesized in the ER and is subsequently trafficked to other cellular compartments [36].

Additionally, we noticed that some of the AlkEA signal was punctate (Figure 2D). Given that PE is conjugated to LC3 during autophagosome formation, we hypothesized that these AlkEA puncta might represent LC3‐positive vesicles. Accordingly, superresolution Airyscan confocal microscopy confirmed that these puncta colocalize with mScarlet‐i‐LC3, with both the AlkEA and LC3 fluorescence showing ring‐like structures (Figure 2D, inset). This result is consistent with the ER being a major source of autophagosome membrane lipids, because AlkEA incorporation into PE must occur in the ER before transport to growing autophagosomes [37, 38, 39, 40, 41].

To test if AlkEA is incorporated into PE via the Kennedy pathway of phospholipid synthesis, we compared cellular labeling in the presence and absence of meclizine, a small molecule inhibitor of the rate‐limiting Kennedy pathway enzyme, PCYT2 (Figure S3A) [42]. Meclizine treatment significantly reduced AlkEA staining (Figure S3B), indicating that probe labeling occurs largely through the Kennedy pathway. We also performed AlkEA labeling in the presence of excess ethanolamine, which resulted in greatly reduced AlkEA incorporation, as seen both by fluorescence microscopy (Figure S4A) and flow cytometry (Figure S4B). Together, these results indicate that AlkEA competes with native ethanolamine as substrate for the Kennedy pathway.

### 2.3 Characterization of AlkEA‐Labeled Lipid Species

To identify the AlkEA‐labeled phospholipid species, we performed Bligh‐Dyer lipid extraction [43] from cells incubated with AlkEA. The resulting lipid extracts were subjected to CuAAC reaction with a fluorophore‐azide prior to HPLC analysis (Figure 3A). To generate synthetic standards of AlkEA‐containing PE, we performed chemoenzymatic reactions using phospholipase D (PLD) from *Arachis hypogaea* to catalyze transphosphatidylation of PC with AlkEA, affording an AlkEA‐labeled PE standard (Figure 3B) [25].

**FIGURE 3:**
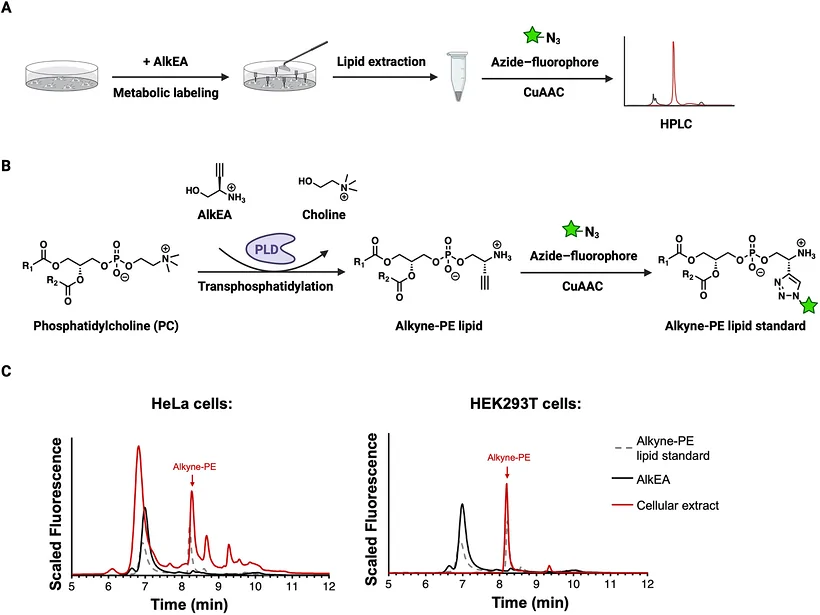
HPLC analysis confirms AlkEA metabolic incorporation into PE. (A) Schematic of HPLC analysis workflow. Cells are incubated with AlkEA (1 mM, 24 h) or vehicle, followed by lipid extraction, CuAAC reaction with BODIPY‐azide, and HPLC analysis. (B) An alkyne‐PE lipid standard was generated via PLD‐mediated transphosphatidylation, in which the choline headgroup of PC 36:1 is replaced by AlkEA. The alkyne handle is then functionalized with BODIPY‐azide for fluorescence detection by HPLC. *R*_1_ = 18:1 (*sn*‐1 acyl chain); *R*_2_ = 18:0 (*sn*‐2 acyl chain). (C) HPLC chromatograms of lipid extracts from HeLa and HEK 293T cells labeled with AlkEA (1 mM, 24 h), following CuAAC reaction with BODIPY‐azide. The peak at ∼8 min corresponds to BODIPY‐derivatized PE, confirmed by the retention time matching that of the derivatized alkyne‐PE standard.

Both the cellular lipids and the lipid standard were subjected to CuAAC reaction with BODIPY‐azide, to produce BODIPY‐tagged PE as a standard for HPLC analysis. For the cellular lipid samples, we observed peaks with retention times matching the alkynyl PE standard, suggesting that AlkEA was incorporated into an alkyne‐containing PE species. Both HeLa and HEK 293T cells incorporated AlkEA into PE, with HEK 293T showing stronger labeling, with no detectable residual AlkEA (Figure 3C). As expected, ethanolamine competition revealed a large decrease in the AlkEA‐PE signal (Figure S4C). Together, these findings further support that AlkEA is incorporated into PE through the Kennedy pathway, preserving the alkyne handle for downstream click chemistry derivatization.

We next performed LC–MS analysis of cellular lipid extracts, to demonstrate the presence of alkynyl‐PE species. To circumvent mass overlap between alkynyl‐PE and abundant endogenous PE species, we conjugated azidopropanol to the alkyne moiety via CuAAC, generating a diagnostic mass shift that unambiguously distinguishes alkynyl‐PE from natural PE (Figure 4A). Encouragingly, azidopropanol‐tagged alkynyl‐PE species were detected across a diverse range of acyl chain compositions, with the overall lipid profile closely mirroring that of endogenous PE (Figure 4B,C). Importantly, AlkEA treatment did not perturb the endogenous PE lipidome, as indicated by the similar abundance and acyl chain composition of endogenous PE species in AlkEA‐labeled and control cells (Figure 4C). Notably, we did not observe peaks corresponding to alkynyl‐PC, suggesting that AlkEA‐incorporated lipids are not substrates for the PEMT‐mediated methylation pathway that converts PE to PC. Collectively, these results demonstrate that AlkEA is predominantly incorporated into PE.

**FIGURE 4:**
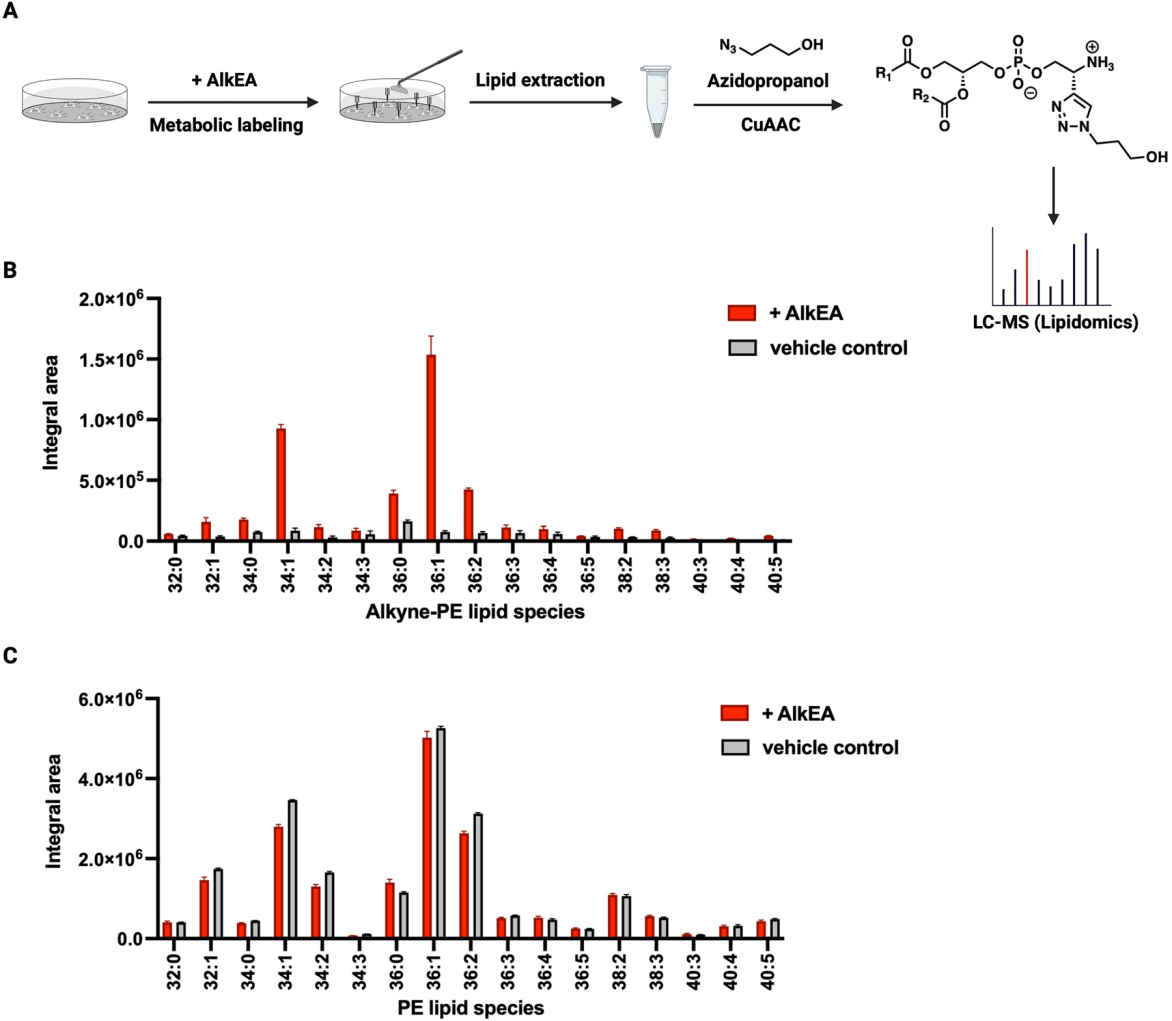
LC–MS analysis of AlkEA metabolic incorporation into PE species. (A) Schematic of LC–MS analysis workflow. HEK 293T cells are incubated with AlkEA (1 mM, 24 h) or vehicle, followed by lipid extraction, CuAAC reaction with azidopropanol, and LC–MS analysis. *R*_1_, *R*_2_ = fatty acyl chains. (B) LC–MS analysis of alkyne‐PE species identified by acyl chain composition (chain length:degree of unsaturation) and quantified by peak area. (C) As in (B) but showing quantification of endogenous PE species. Data are presented as mean ± SD (*n* = 3).

### 2.4 AlkEA Labeling of PE‐Derived Posttranslational Protein Modifications

Finally, we tested whether AlkEA labels proteins modified with ethanolamine moieties, including PE‐conjugated proteins and GPI anchors (Figure 5A). Proteins of the ATG8 family (Atg8 in yeast and LC3/GABARAP in mammals) are modified at C‐terminal glycine residues as amides with the primary amine of PE (Figure 5B); this PE conjugation serves to anchor ATG8 proteins to autophagosomal membranes, enabling the recruitment of autophagy receptors and cargo, to facilitate autophagosome maturation and subsequent cargo degradation [11]. Motivated by imaging data showing colocalization of the AlkEA signal with autophagosomal membranes (Figure 2D), we hypothesized that AlkEA might label LC3‐II, the PE‐conjugated form of LC3. Lysates of AlkEA‐labeled cells were subjected to CuAAC reaction with biotin‐azide, and biotinylated proteins were affinity isolated on streptavidin beads, followed by SDS‐PAGE and immunoblotting. Whereas LC3 appears as two bands in the input samples, corresponding to unconjugated (LC3‐I) and PE‐conjugated (LC3‐II) species, only LC3‐II was present in the streptavidin pull‐down, indicating that the PE‐conjugated form was selectively recovered from AlkEA‐labeled samples (Figure 5C). Importantly, ethanolamine competition or Kennedy pathway inhibition abolished AlkEA incorporation into LC3‐II. Conversely, Bafilomycin A1, which blocks autophagic degradation and causes an accumulation of LC3‐positive autophagosomes, led to a marked increase in AlkEA‐labeled LC3‐II levels (Figure 5C).

**FIGURE 5:**
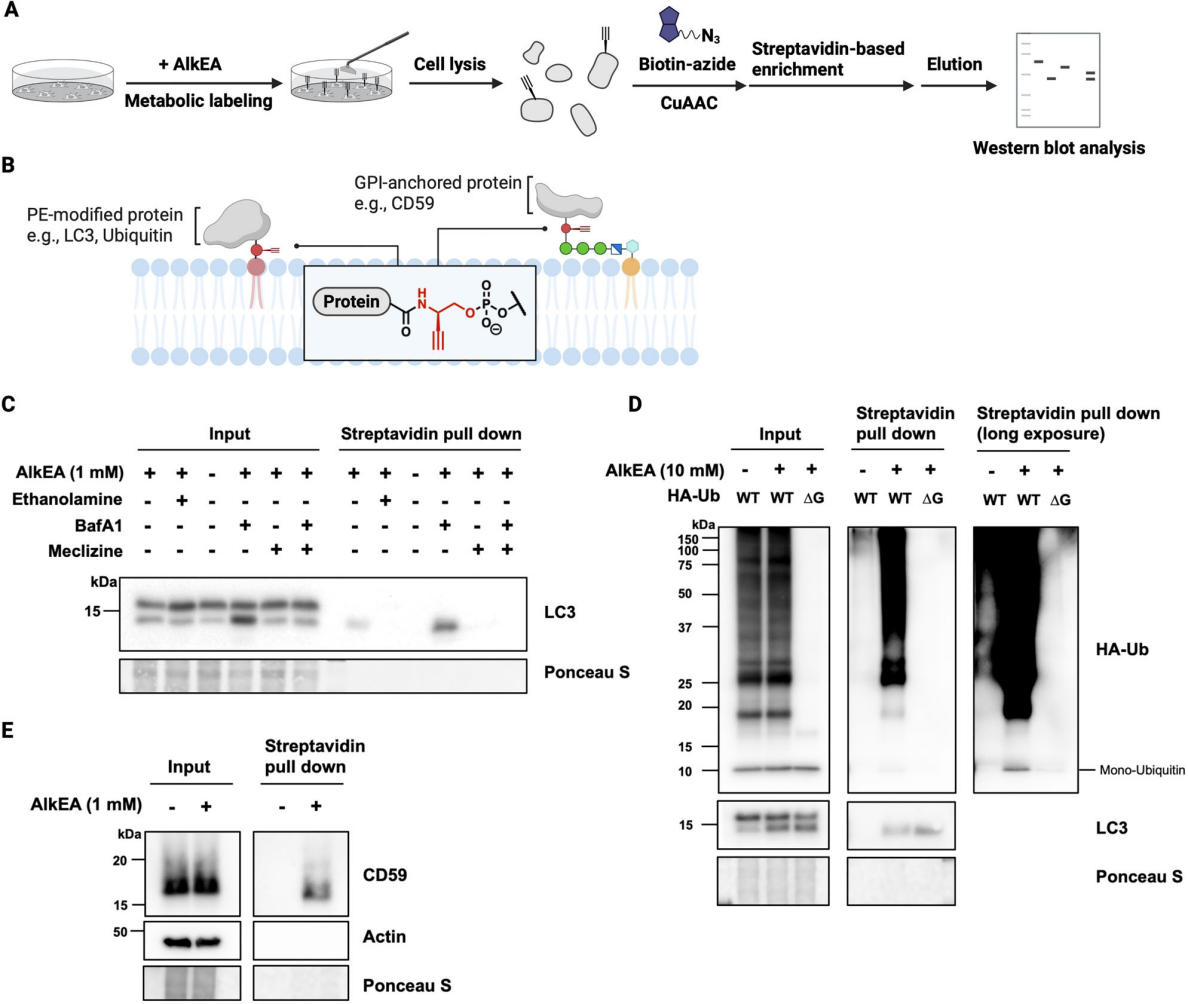
Probing PE‐dependent posttranslational protein modifications using AlkEA. (A) Schematic of affinity enrichment workflow. HEK 293T cells are incubated with AlkEA for 24 h, followed by cell lysis, CuAAC reaction with biotin‐PEG_3_‐azide, streptavidin affinity enrichment, and immunoblotting. (B) Schematic of AlkEA incorporation into PE‐conjugated proteins (e.g., LC3, ubiquitin) and GPI‐anchored proteins (e.g., CD59). (C) Whole cell lysates (input) and streptavidin‐enriched fractions were analyzed by SDS‐PAGE followed by immunoblotting for LC3. Ponceau S staining of the membrane serves as loading control. Ethanolamine (10 mM) was used to compete AlkEA metabolic incorporation. Meclizine (40 µM) was used to block the Kennedy pathway. Bafilomycin A1 (BafA1, 100 nM) was added 6 h prior to harvesting. (D) As in (C), but with cells expressing the indicated HA‐tagged ubiquitin constructs (wild type or a C‐terminal deletion mutant that cannot be conjugated, Δ*G*), and immunoblotting for HA and LC3. (E) As in (C), but with immunoblotting for CD59 and actin (loading control).

We next examined whether ubiquitin, which was recently discovered as a PE‐modified protein, can be labeled by the same approach [10]. Although it required a higher concentration of AlkEA, we detected AlkEA conjugated to HA‐tagged mono‐ubiquitin (Figure 5D). Importantly, removal of the C‐terminal glycine residue required for ubiquitin conjugation completely abolished mono‐ubiquitin attachment to AlkEA, further confirming the specificity of the observed modification.

Finally, we asked if AlkEA labels GPI anchors, which include a phosphoethanolamine moiety that forms an amide bond with the C‐terminus of the anchored protein, as well as a phosphoethanolamine moiety attached to one of the sugar residues (Figure 5B) [8]. As shown in Figure 5E, AlkEA was incorporated into CD59, a representative GPI‐anchored protein. Collectively, these results demonstrate that AlkEA is versatile metabolic label for ethanolamine‐containing posttranslational protein modifications.

## 3 Conclusion

We developed a physiological alkynyl analog of ethanolamine, for metabolic labeling of PE and PE‐derived posttranslational modifications. Subsequent derivatization via click chemistry allows microscopic imaging of cellular PE, as well as affinity isolation of proteins conjugated to PE (such as ubiquitin and LC3) or modified with PE‐derived phosphoethanolamine groups (such as GPI‐anchored proteins).

Our study provides the first example of introducing a bioorthogonal handle on the lipid moiety of LC3‐II through metabolic labeling, representing a new approach to study autophagosome formation and maturation. The successful pull‐down of PE‐conjugated ubiquitin is also significant, as the biological functions of this recently identified ubiquitin modification remain unexplored; we envision that AlkEA could enable the comprehensive identification of proteins tagged with PE‐conjugated ubiquitin. Finally, AlkEA labeling of GPI‐anchored proteins further demonstrates the broad utility of this probe across distinct ethanolamine‐containing lipid modifications.

Beyond protein labeling, we envision that AlkEA metabolic labeling can be combined with organelle‐targeted dyes to investigate the spatiotemporal dynamics of inter‐organelle PE transport [44, 45, 46]. The alkyne moiety could be replaced with other sufficiently small groups such as diazirine‐containing bifunctional tags, for photoaffinity‐based capture of PE‐interacting proteins [47, 48, 49]. Our bioorthogonal approach for labeling PE opens up numerous possibilities for dissecting the diverse roles of PE in membrane organization, autophagy, and protein modification.

## Author Contributions

C.Y.J. and A.S. conceived of the project, C.Y.J. synthesized AlkEA analogs, and C.Y.J. and A.S. characterized AlkEA incorporation into PE. B.M.W., Y.C., Z.X., and J.M.B. designed studies to fully characterize AlkEA use for PE labeling and PE conjugation. B.M.W. and Y.C. carried out PE metabolic labeling studies, Y.C. carried out PE conjugation studies. Y.C. and J.M.B. wrote the manuscript and received input from all authors.

## Conflicts of Interest

The authors declare no conflicts of interest.

## Supporting information




**Supporting File**: anie74034‐sup‐0001‐SuppMat.pdf.

## Data Availability

The data that supports the findings of this study are available in the Supporting Information of this article
