# Supplementary material for: Bioorthogonal Tools for Ethanolamine Lipids and Protein Conjugates
Source: Angew Chem Int Ed Engl. 2026 Aug 6;65(40):e1866870. doi: 10.1002/anie.1866870 (PMC13520642; doi:10.1002/anie.1866870)
Supplement: Supplementary file 1 [file ANIE-65-e1866870-s001.pdf]

Supporting Information for

Bioorthogonal Tools for Ethanolamine Lipids and Protein Conjugates

Yuan-Ting Cho[1,2], Cindy Y. Jao[3], Zijun Xia[1,2], Brittany M. White-Mathieu[4*], Adrian Salic[3*], Jeremy M. Baskin[1,2*]

[1]Weill Institute for Cell and Molecular Biology, Cornell University, Ithaca, New York 14853, USA
[2]Department of Chemistry and Chemical Biology, Cornell University, Ithaca, New York 14853, USA
[3]Department of Cell Biology, Harvard Medical School, Boston, MA 02115, USA
[4]Department of Chemistry, University of New Hampshire, Durham, NH 03820, USA

* Correspondence: jeremy.baskin@cornell.edu, asalic@hms.harvard.edu, Brittany.WhiteMathieu@unh.edu

Contents

Figures S1–S4
Materials and Methods
Synthetic Methods
NMR Spectra
References

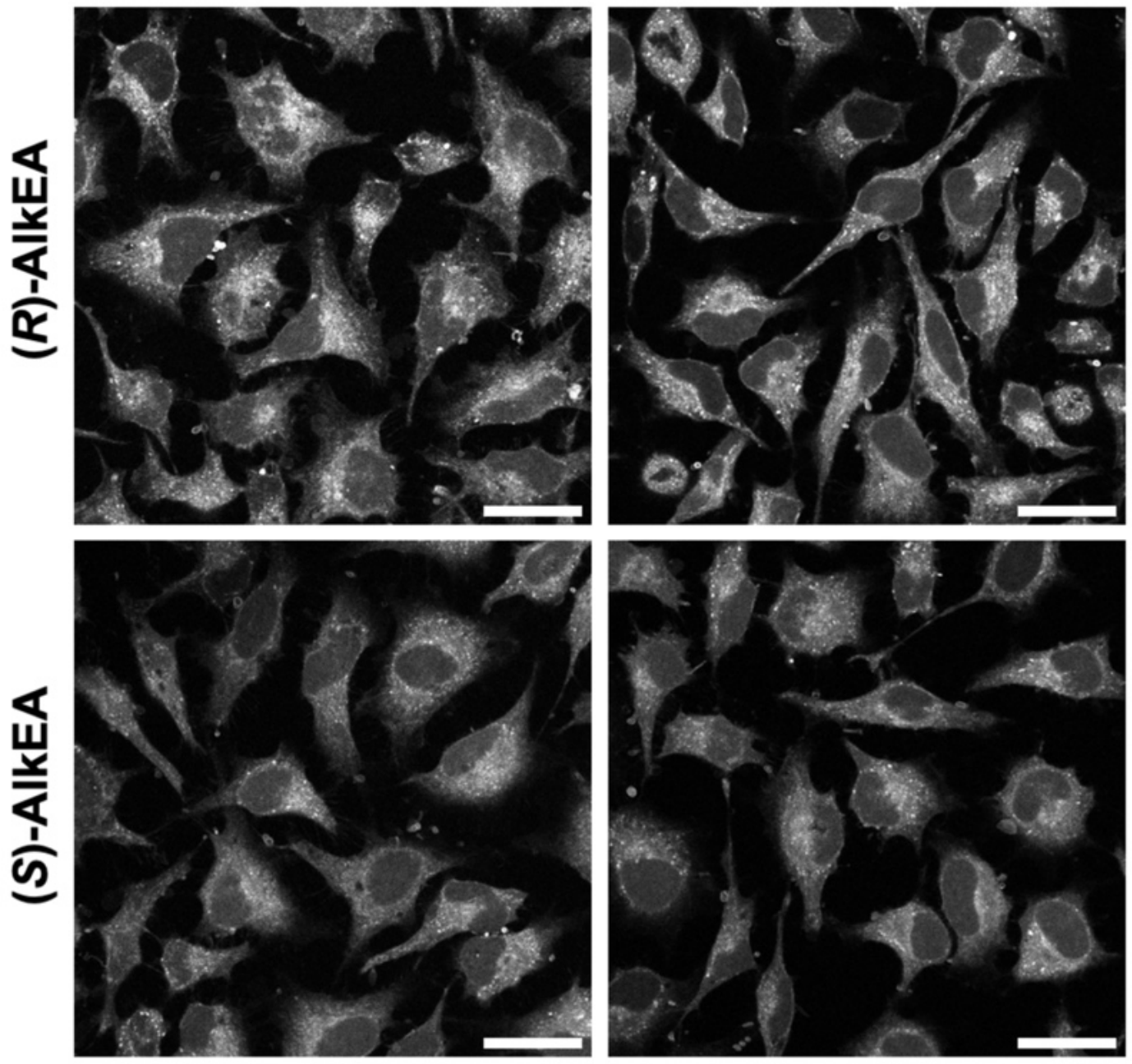

Figure S1. (*R*)-AlkEA exhibits stronger membrane-localized fluorescence signal than (*S*)-AlkEA. HeLa cells were treated with (*R*)- or (*S*)-AlkEA (1 mM, 24 h), fixed with 4% paraformaldehyde, and subjected to CuAAC conjugation with BODIPY-FL-azide. Representative confocal fluorescence images are shown. Scale bar, 30 µm.

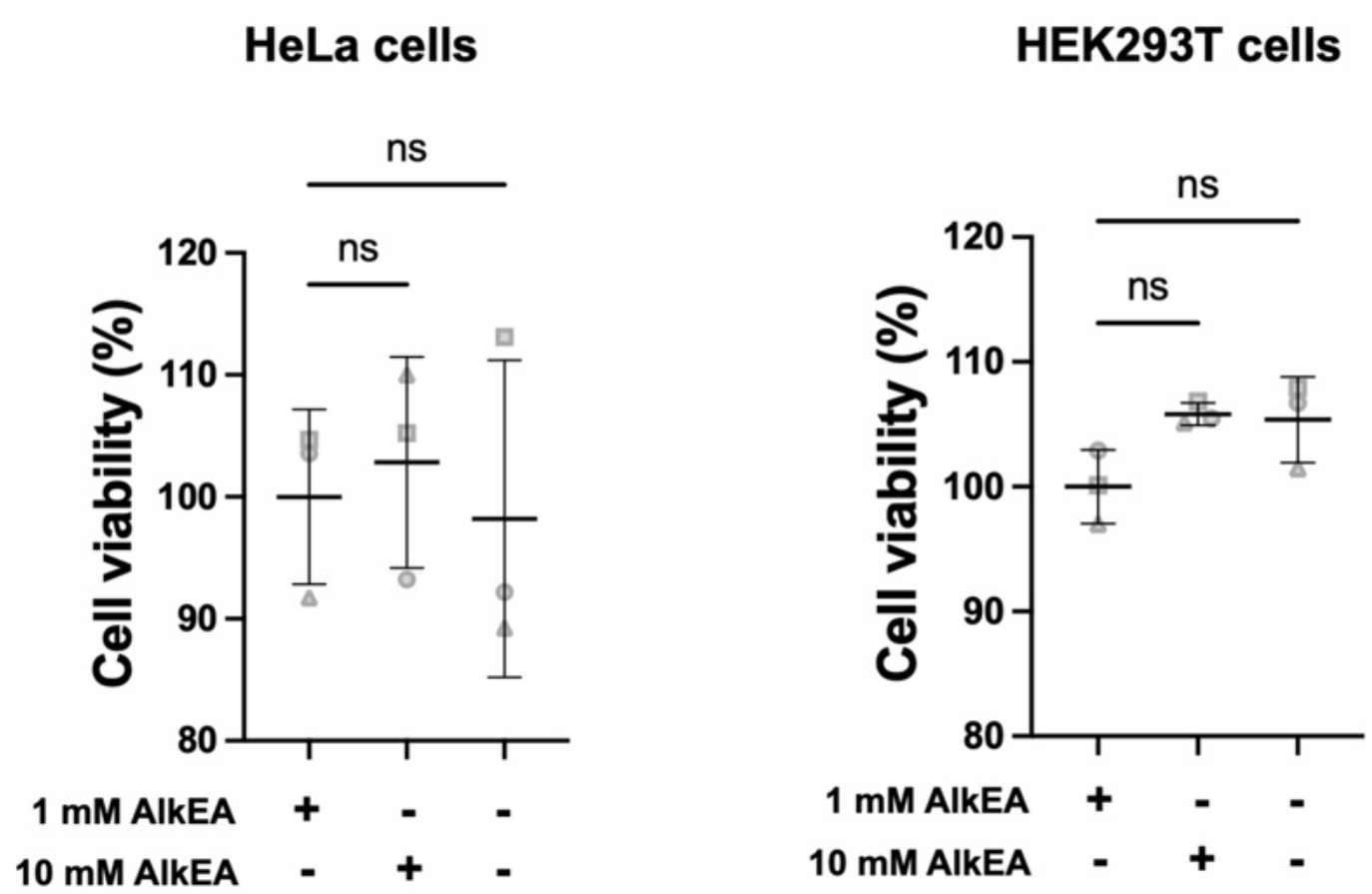

Figure S2. Cell viability of HeLa and HEK293T cells following AlkEA treatment. Cells were treated with AlkEA at the indicated concentrations for 24 h, and viability was determined by MTT assay. Data are presented as mean ± SD. Statistical significance was determined by one-way ANOVA with Tukey's post-hoc test (n = 3; ns, not significant).

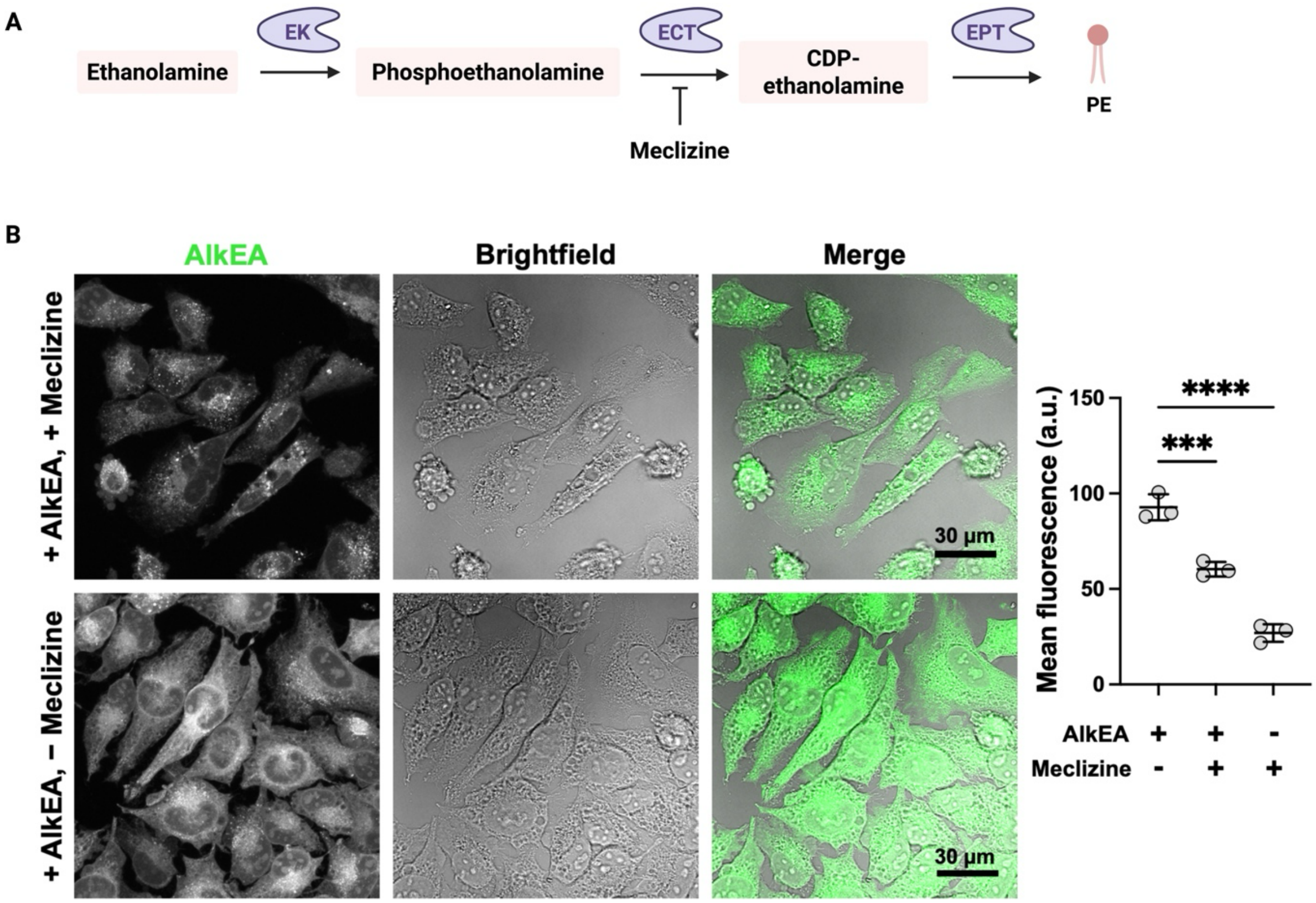

Figure S3. Meclizine treatment reduces AlkEA incorporation into cellular membranes. (A) Meclizine inhibits PCYT2 (phosphate cytidylyltransferase 2), blocking the conversion of phosphoethanolamine to CDP-ethanolamine in the Kennedy pathway. (B) HeLa cells were treated with AlkEA (1 mM) in the presence or absence of meclizine (50 µM) for 24 h. Following AlkEA treatment, cells were fixed with 4% paraformaldehyde and conjugated with BODIPY-azide prior to confocal imaging. Fluorescence intensity of individual cells in view were quantified from maximum intensity projection images and presented as mean ± SD. Statistical significance was determined by one-way ANOVA with Tukey's post-hoc test (n = 3; **** $p < 0.0001$; *** $p = 0.0007$). a.u.: arbitrary units.

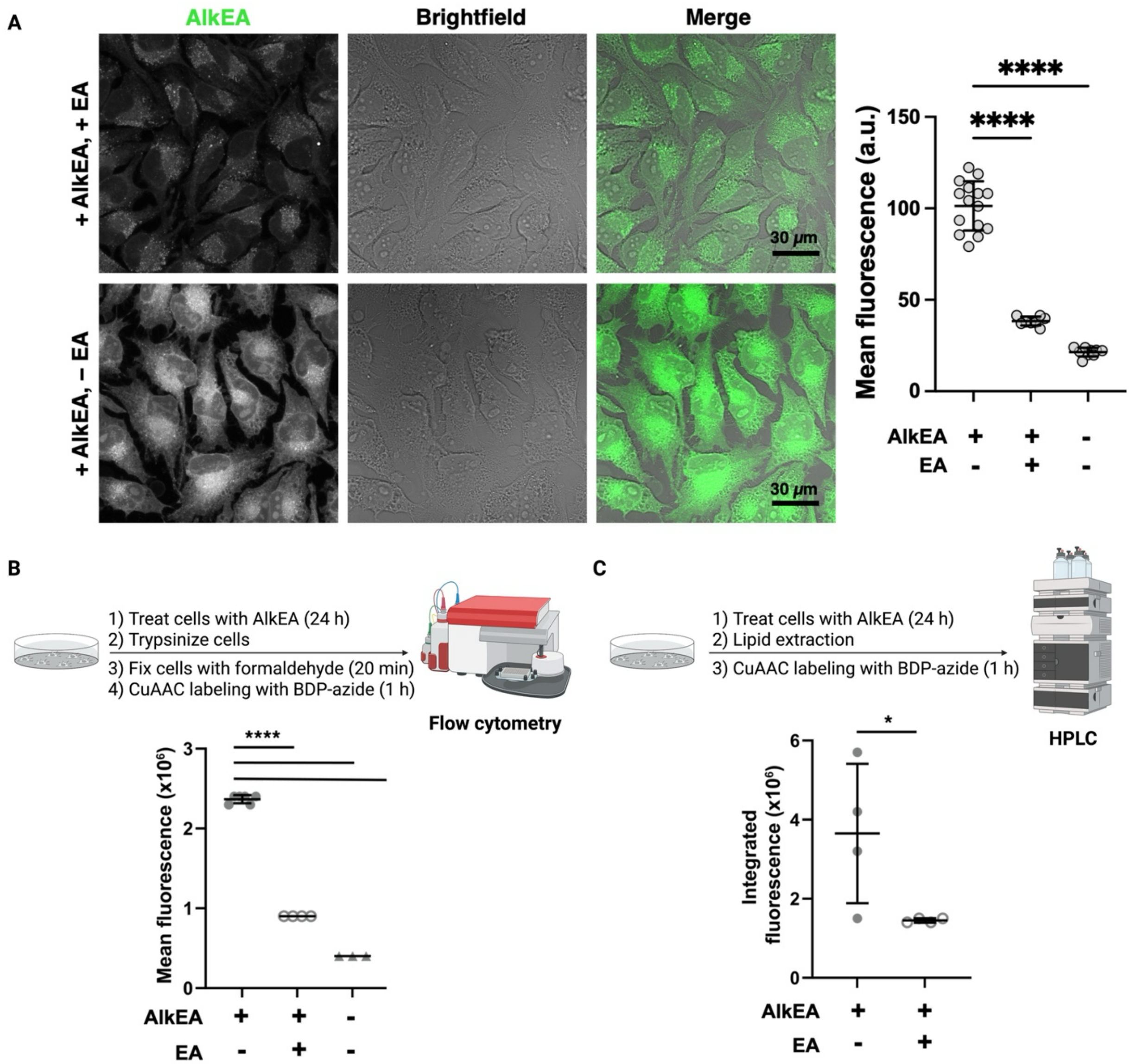

Figure S4. Ethanolamine competition reduces AlkEA-dependent labeling. (A) HeLa cells were treated with AlkEA (1 mM) in the presence or absence of ethanolamine (10 mM) for 24 h. Following AlkEA treatment, cells were fixed with 4% paraformaldehyde and conjugated with BODIPY-azide prior to confocal imaging. Fluorescence intensity was quantified from maximum intensity projection images and presented as mean ± SD. (B) HeLa cells were treated with AlkEA (1 mM) in the presence or absence of ethanolamine (10 mM, pH 7.6) for 24 h. Following AlkEA treatment, cells were fixed with 4% paraformaldehyde, conjugated with BODIPY-azide, and analyzed by flow cytometry. (C) HeLa cells were treated with AlkEA (1 mM) in the presence or absence of ethanolamine (10 mM) for 24 h. Lipids were then extracted, subjected to CuAAC conjugation with BODIPY-azide, and analyzed by HPLC. Statistical significance was determined by one-way ANOVA with Tukey's post-hoc test ($n = 3$; **** $p < 0.0001$; * $p < 0.05$). a.u.: arbitrary units.

MATERIALS AND METHODS

General experimental details

Unless otherwise noted, reactions were carried out under an inert nitrogen atmosphere using standard Schlenk technique. All reagents used were analytical grade and obtained from commercial suppliers without further purification. Dulbecco's modified Eagle medium (DMEM), fetal bovine serum (FBS), penicillin/streptomycin (P/S), 100 mM sodium pyruvate, phosphate-buffered saline (PBS), and 0.05% trypsin-EDTA (trypsin) were purchased from Corning. Lipofectamine 2000 was purchased from Thermo Fisher. 3-Azido-1-propanol was purchased from Sigma-Aldrich. BDP-FL-azide was purchased from Lumiprobe. Biotin-PEG3-azide and THPTA were purchased from Click Chemistry Tools. Silica gel purification was performed with 60 Å, 40-63 µm SiliCycle SiliaFlash irregular silica gel. Thin layer chromatography (TLC) was performed using EMD Millipore Silica gel 60 F254 plates for normal phase chromatography and developed plates were visualized visualized by treatment with ceric ammonium molybdate (CAM) or KMnO4 stains. NMR spectra were recorded on a Bruker 500 MHz spectrometer with a BBO H&F cryoprobe at ambient temperature. 1H NMR spectra were obtained in CDCl3 (referenced to CHCl3, δ 7.24 ppm) or MeOD (referenced to MeOH, δ 3.31 ppm), and all 13C NMR spectra were taken in CDCl3 (referenced to CDCl3, δ 77.00 ppm) or MeOD (referenced to MeOH, δ 49.15 ppm). LC-MS analysis was performed on an Agilent 6230 electrospray ionization-time-of-flight (ESI-TOF) MS coupled to an Agilent 1260 HPLC equipped with an Agilent InfinityLab Poroshell 120 EC-C18 reverse phase column (3.0 x 50 mm, 2.7 µm). HPLC analysis was performed on a Shimadzu LC-20AR HPLC equipped with an SPD20AV UV/Vis detector, an RF-20A fluorescence detector, and a Phenomenex Luna silica 3-µm, 100-Å, 25-cm × 4.6-mm column. Confocal microscopy was performed on a Zeiss LSM 800 confocal laser scanning microscope equipped with an incubation system for temperature control and CO2/moisture stable supply, a Plan Apochromat 40X 1.4 NA oil immersion objective oil immersion objective, 405, 488, 561, and 640 nm solid-state lasers, and two GaAsP PMT detectors. Images were acquired using Zeiss Zen Blue 2.3 and analyzed using ImageJ/FIJI[1]. Flow cytometry was performed on a BD Accuri C6 flow cytometer, and analysis was performed using the BD Accuri C6 analysis software.

Cell culture

HeLa (ATCC, CRM-CCL-2) and HEK293T (ATCC, CRL-3216) cells were cultured at 37 °C in a humidified atmosphere containing 5% CO_2. HeLa cells were maintained in DMEM supplemented with 10% FBS and 1% penicillin-streptomycin. HEK293T cells were maintained in DMEM supplemented with 10% FBS, 1% penicillin-streptomycin, and 1 mM sodium pyruvate. Cells were passaged at densities between 1×10^5 and 1.6×10^6 cells/mL.

Plasmids and cloning

mCherry-PH(OSBP) was previously generated in our lab[2]. mScarlet-i-LC3 was generated by subcloning LC3 from pMRX-IP-GFP-LC3-RFP into the mScarlet-i-C1 vector. mCherry-OMP25TM, mRFP-Sec61β, and iRFP-PH(OSBP) were kindly provided by P. De Camilli (Yale University, New Haven, CT). pMRX-IP-GFP-LC3-RFP was purchased from Addgene (plasmid #84573).

Antibodies

Anti-LC3 (D3U4C), rabbit mAb, Cell Signaling Technology (12741); Anti-HA antibody (3F10), rat mAb, Millipore Sigma (11867423001); Anti-CD59 (YTH53.1), mouse mAb, Santa Cruz (sc-59095); Anti-actin (AC-40), mouse mAb, Sigma-Aldrich (A4700).

Transient transfection of mammalian cells

35-mm glass-botton dishes (14 mm diameter, #1.5 thickness, Matsunami Glass) were treated with poly-L-lysine (0.1 mg/ml) in PBS for 35 min in a cell incubator. The solution was aspirated and the dishes were rinsed once with PBS. HeLa and HEK293T cells were then seeded at 15000 cells per dish 12 h prior to transfection. Cells were transfected with the indicated plasmids using Lipofectamine 2000 (Invitrogen) according to the manufacturer's instructions. Cells were incubated with the transfection mixture in Opti-MEM (Corning) supplemented with 10% FBS for 7-8 h, after which the medium was replaced with regular growth medium containing AlkEA or vehicle (24 h) for labeling experiments.

Metabolic labeling with AlkEA

HeLa cells were seeded at 15000 cells per 35-mm dish 12 h prior to the experiment. Cells were grown overnight, after which the medium was aspirated and replaced with growth medium containing 1 mM AlkEA (prepared from a 1 M solution in water) or regular growth medium as vehicle controls. Cells were incubated in the labeling media or regular media for 24 h. For Kennedy pathway inhibition, meclizine (40 µM or 50 µM, prepared from a 80 mM solution in DMSO) was added to the growth medium simultaneously with AlkEA treatment. For competition experiments, ethanolamine (10 mM, pH 7.6, prepared from a 10 M solution in water) was added to the growth medium simultaneously with AlkEA.

Confocal microscopy and flow cytometry

Following metabolic labeling, the medium was aspirated, and cells were washed three times with PBS, fixed with 4% paraformaldehyde (1 mL per dish, 20 min, room temperature). The fixative was aspirated and quenched with 100 mM glycine in PBS (1 mL per dish) for 10 min at room temperature. Cells were washed three times with Tris-buffered saline (TBS) and labeled with 100 µL of freshly prepared CuAAC solution for 1 h at room temperature in the dark. The CuAAC solution was prepared immediately before use by sequential addition of the following reagents: 100 mM Tris (pH 8.5), 5 µM BODIPY-FL-azide or Alexa Fluor 488-azide (prepared from a 5 mM stock in DMSO), 1 mM $CuSO_4$ (prepared from a 20 mM stock in water), and 50 mM sodium ascorbate (prepared from a 500 mM stock in water). Following incubation, the CuAAC solution was aspirated and cells were washed sequentially with TBS (three times), 500 mM NaCl (three times), and TBS (three times). Cells were immediately imaged by confocal microscopy. Multicolor images were acquired in two-channel line-switching mode, with z-stacks collected at 0.45 µm intervals. For flow cytometry analysis, cells were labeled as described above and resuspended in PBS supplemented with 0.5% FBS following the final wash. Samples were kept on ice and analyzed by flow cytometry. Cells were gated on a minimum of 10,000 live cells as determined by forward scatter/side scatter (FSC/SSC) analysis.

Lipid extraction

5×10^5 cells were seeded in a 60-mm dish and allowed to grow overnight. Cells were treated under the indicated conditions as described above. The treatment medium was aspirated, and cells were washed twice with cold PBS (1 mL per wash). Lipids were extracted using a

modified Bligh-Dyer procedure. Briefly, 250 µL of cold methanol, 125 µL of cold 20 mM acetic acid, and 100 µL of cold PBS were added sequentially to the dish. Cells were scraped from the dish and transferred to 1.5 mL microcentrifuge tubes. 250 µL of chloroform was added to the suspension, and the mixture was vortexed for 1 min and centrifuged at 16,000 x g for 2 min. The organic (lower) phase was carefully removed and transferred to a new microcentrifuge tube. A second extraction was performed by adding chloroform (250 µL) to the remaining aqueous phase, followed by vortexing and centrifugation as above. The two organic phases were combined and dried under a stream of nitrogen. The lipid extract was stored at -20 ºC until further use.

HPLC analysis

Lipid extracts for HPLC fluorescence detection were subjected to CuAAC conjugation with BODIPY-FL-azide. Briefly, dried lipid films were resuspended in 7 µL of chloroform, combined with 30 µL of CuAAC reaction master mix, and incubated at 42 ºC for 5 h in the dark. The master mix was prepared by combining 60 µL of [acetonitrile]$_4$CuBF$_4$ (10 mM in degassed acetonitrile), 240 µL of degassed ethanol, and 2.6 µL of BODIPY-FL-azide (10 mM in DMSO). Following incubation, the reaction mixture was diluted with 63 µL of chloroform:methanol (9:1, v/v), filtered, and analyzed by HPLC. Analysis was performed by normal-phase HPLC using a binary gradient elution system. Solvent A was chloroform:methanol:ammonium hydroxide (95:7:0.5, v/v/v) and solvent B was chloroform:methanol:water:ammonium hydroxide (60:34:5:0.5, v/v/v/v). Separation was achieved using a linear gradient from 100% A to 100% B over 10 min.

LC-MS analysis

Lipid extracts for LC-MS detection were subjected to CuAAC conjugation with 3-azidopropan-1-ol. Briefly, dried lipid films were resuspended in 7 µL of chloroform, combined with 30 µL of CuAAC reaction master mix, and incubated at 42 ºC for 5 h. The master mix was prepared by combining 60 µL of [acetonitrile]$_4$CuBF$_4$ (10 mM in degassed acetonitrile), 240 µL of degassed ethanol, and 1 µL of neat 3-azidopropan-1-ol. Following incubation, the reaction mixture was diluted with 63 µL of chloroform:methanol (9:1, v/v), filtered, and analyzed by LC-MS. Analysis was performed by reverse phase HPLC using a binary gradient elution system where solvent A was methanol: acetonitrile: water (1:1:3, v/v/v) supplemented with 5 mM ammonium acetate and solvent B was isopropanol supplemented with 5 mM ammonium acetate. Separation was achieved using a linear gradient from 100% A to 100% B over 20 min at a flow rate of 0.3 ml/min. Conjugated lipids were detected using a dual AJS ESI source operating in negative mode, acquiring in extended dynamic range over m/z 100-1700 at one spectra per second; gas temperature, 200 °C; drying gas flow, 12 L/min; nebulizer pressure, 35 psig; fragmentor voltage, 300 V; nozzle voltage, 500 V.

Streptavidin enrichment of AlkEA-labeled proteins

HEK293T cells were labeled with AlkEA as described above, using 1 mM for detection of LC3 and CD59, or 10 mM for detection of HA-ubiquitin. The following compounds were added at the indicated concentrations: meclizine (40 µM, 24 h), ethanolamine (10 mM, 24 h), and bafilomycin A1 (BafA1, 100 nM, 6 h before harvesting). Following metabolic labeling, the medium was aspirated and dishes were washed twice with cold PBS. Cells were lysed by addition of 500 µL cold RIPA buffer supplemented with 1x cOmplete protease inhibitor cocktail and scraped from the dish. The lysate was homogenized by probe sonication (20% amplitude, 4 s) and

clarified by centrifugation at 17,000 x g for 10 min at 4 °C. Protein concentration was determined using the Pierce BCA Protein Assay Kit (Thermo Fisher Scientific) according to the manufacturer's instructions.

From the whole cell lysate prepared as described above, 1.3 mg of total protein was transferred to a new 1.5 mL microcentrifuge tube and brought to a total volume of 500 µL with cold RIPA buffer supplemented with 1x cOmplete EDTA-free protease inhibitor cocktail. Pre-washed streptavidin-agarose beads (10 µL; Thermo Fisher Scientific, cat. no. 20359) were added to the lysate, and the suspension was rotated end-over-end at room temperature for 2 h. The beads were pelleted by centrifugation, and the supernatant was collected and subjected to CuAAC conjugation at room temperature for 1 h in the dark. The CuAAC reaction mixture was prepared by sequential addition of the following reagents: $CuSO_4$ (1 mM, prepared from a 20 mM aqueous stock), THPTA (600 µM, prepared from a 12 mM aqueous stock; Click Chemistry Tools), biotin-PEG_3-azide (100 µM, prepared from a 10 mM aqueous stock; Click Chemistry Tools), and sodium ascorbate (50 mM, prepared from a 500 mM aqueous stock).

Following the CuAAC reaction, proteins were precipitated by addition of cold methanol (400 µL), chloroform (100 µL), and water (200 µL). The mixture was vortexed briefly and centrifuged at 17,000 x g for 8 min at 4 °C. The upper aqueous layer was discarded, and 1 mL of cold methanol was added, followed by centrifugation at 17,000 x g for 3 min at 4 °C. The supernatant was carefully discarded, and the protein pellet was resuspended in 200 µL of 1.2% SDS in PBS by probe sonication (20% amplitude, 4 s). The precipitation and resuspension were repeated once to ensure complete removal of CuAAC reagents. The remaining lysate was diluted with 1 mL of PBS supplemented with 1X cOmplete EDTA-free protease inhibitor cocktail to reduce the final SDS concentration to 0.2%. Pre-washed streptavidin-agarose beads (15 µL) were added to the diluted lysate, and the suspension was rotated end-to-end at room temperature for 2 h. The beads were pelleted by centrifugation at 1000 x g for 2 min, the supernatant was discarded, and the beads were washed five times with 0.2% SDS in PBS (1 mL per wash). Finally, the beads were resuspended in 20 µL of 2X SDS loading buffer and heated at 95 °C for 5 min to elute enriched proteins for SDS-PAGE analysis.

<u>SDS-PAGE and Western blot</u>

Whole cell lysate input and streptavidin-enriched samples were resolved by SDS-PAGE on 15% polyacrylamide gels in running buffer (25 mM Tris, 96 mM glycine, 0.1% SDS) at 160 V for 1 h. After SDS-PAGE running, proteins were transferred to methanol-activated PVDF membranes using the Trans-Blot Turbo transfer system (Bio-Rad) in 1X Turbo transfer buffer (Bio-Rad, cat. no. 10026938) at 25 V and 1.3 A for 10 min. Membranes were blocked in 5% non-fat milk in TBS-T for 1 h at room temperature, then washed three times with TBS-T (5 min per wash). Primary antibodies diluted in 5% non-fat milk in TBS-T were incubated with membranes overnight at 4 °C. Following primary antibody incubation, membranes were washed three times with TBS-T (7 min per wash) and incubated with the appropriate HRP-conjugated secondary antibody diluted in freshly prepared 5% non-fat milk in TBS-T for 1 h at room temperature. Membranes were then washed three times with TBS-T (7 min per wash) and twice with TBS (7 min per wash). Chemiluminescence was developed using Clarity or Clarity Max ECL Western Blotting Substrate (Bio-Rad) for 5 min and imaged on a ChemiDoc Imaging System (Bio-Rad).

SYNTHETIC METHODS

*Tert-butyl (R)-4-ethynyl-2,2-dimethyloxazolidine-3-carboxylate (**3**)*

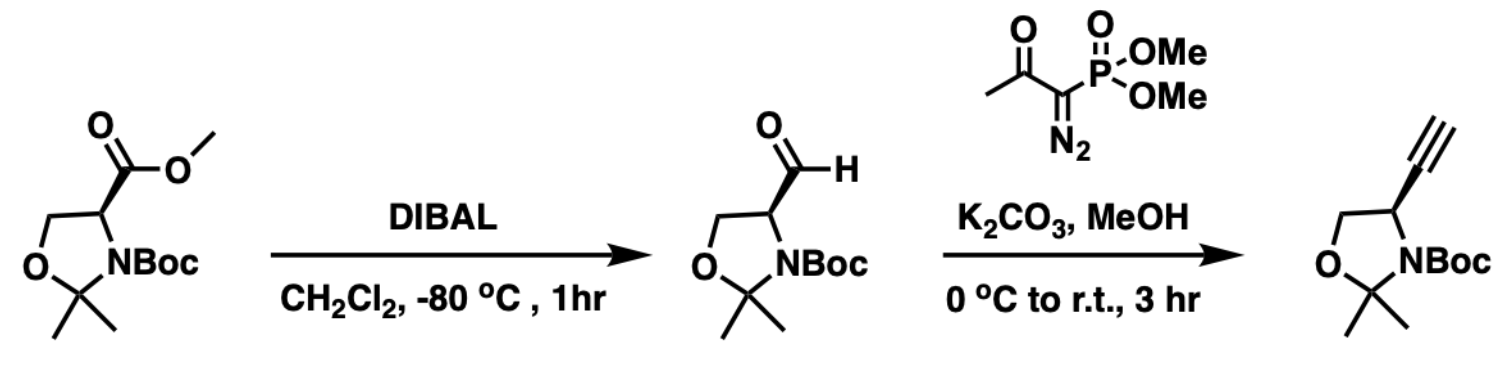

1 2 3

Compound **3** was prepared according to literature procedures[3]. Briefly, to a solution of compound **1** (5 g, 19.28 mmol) in dry CH_2Cl_2 (43 mL) was added DIBAL-H (32.80 mL, 32.80 mmol, 1M in THF) dropwise at -80 °C. The reaction mixture was stirred at -80 °C for 1 h, then quenched with saturated aqueous sodium potassium tartrate. The mixture was warmed to room temperature and stirred for 2 h. The aqueous layer was extracted with CH_2Cl_2 three times. The combined organic layers were washed with brine, dried over anhydrous $MgSO_4$, filtered, and concentrated under reduced pressure to afford compound **2** as a colorless oil.

To a solution of dimethyl (1-diazo-2-oxopropyl)phosphonate (Bestmann-Ohira reagent, 3.50 ml, 23.14 mmol) and crude compound **2** in anhydrous methanol (50 mL) was added K_2CO_3 (4.53 g, 32.78 mmol) at 0 °C. The reaction mixture was stirred at 0 °C for 30 min, then warmed to room temperature and stirred for an additional 3 h. 50 mL of saturated aqueous NH_4Cl and 100 mL hexanes were added, the organic layer separated, dried over anhydrous $MgSO_4$, and concentrated. The residue was purified by column chromatography (EtOAc/hexane = 1/20) to give compound **3** as a colorless oil (1.91 g, 44% over 2 steps). ^{1}H NMR (500 MHz, CD_3CN) δ 4.57-4.49 (m, 1H), 4.02 (dd, 1H, J = 8.8, 5.8 Hz), 3.93 (dd, 1H, J = 8.8, 1.85 Hz), 2.55-2.49 (m, 1H), 1.55 (s, 3H), 1.46 (s, 9H), 1.44 (s, 3H); ^{13}C NMR (125 MHz, CD_3CN) δ 152.31, 94.09, 84.25, 80.74, 71.26, 69.41, 49.18, 28.51, 27.62, 26.42, 25.21, 24.30; LC-MS calcd. for $C_{12}H_{20}NO_3$: 226.1443, found: 226.1430 *m/z* $[M + H]^+$.

*(R)-2-aminobut-3-yn-1-ol ((**R)-AlkEA**)*

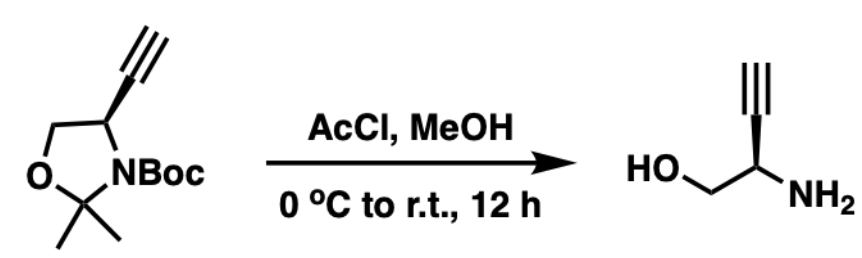

3 (*R*)-AlkEA

(*R*)-AlkEA was prepared according to literature procedures[4]. Briefly, to a solution of compound **3** (1.96 g, 8.70 mmol) in dry methanol (13 mL) was added acetyl chloride (2.5 mL, 34.80 mmol) at 0 °C. The reaction was slowly warmed to room temperature and stirred for 12 h. The crude reaction was concentrated and purified by column chromatography (EtOAc/methanol = 7/3 plus 0.1M ammonia in ethanol) to give **(*R*)-AlkEA** as a yellow solid (0.74 g, 99%). ^{1}H NMR (500 MHz, CD_3OD) δ 4.13 (ddd, 1H, J = 7.6, 4.1, 2.3 Hz), 3.88 (dd, 1H, J = 11.6, 4.1 Hz), 3.70 (dd, 1H, J = 11.6, 7.8 Hz), 3.22 (d, 1H, J = 2.3 Hz); ^{13}C NMR (125 MHz, CD_3OD) δ 78.40, 77.17, 63.08, 45.84; LC-MS calcd. for C_4H_8NO: 86.0606, found: 86.0612 *m/z* $[M + H]^+$.

*Tert-butyl (S)-4-ethynyl-2,2-dimethyloxazolidine-3-carboxylate (**6**)*

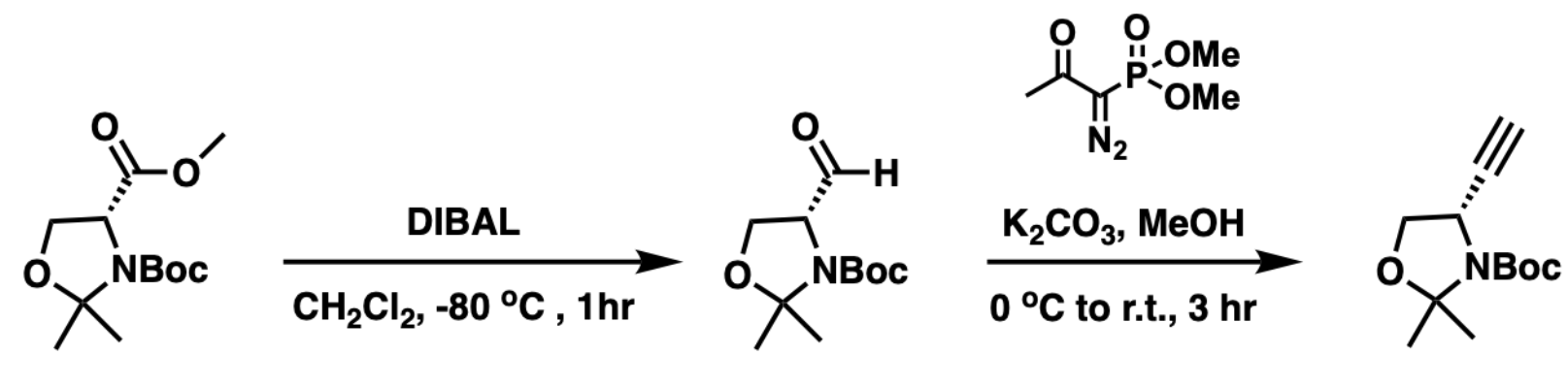

4 5 6

Compound **6** was prepared according to literature procedures[3]. Briefly, to a solution of compound **4** (1 g, 3.86 mmol) in dry CH_2Cl_2 (8.60 mL) was added DIBAL-H (6.56 mL, 6.56 mmol, 1M in THF) dropwise at -80 °C. The reaction mixture was stirred at -80 °C for 1 h, then quenched with saturated aqueous sodium potassium tartrate. The mixture was warmed to room temperature and stirred for 2 h. The aqueous layer was extracted with CH_2Cl_2 three times. The combined organic layers were washed with brine, dried over anhydrous $MgSO_4$, filtered, and concentrated under reduced pressure to afford compound **5** as a colorless oil.

To a solution of dimethyl (1-diazo-2-oxopropyl)phosphonate (Bestmann-Ohira reagent, 1.75 ml, 4.63 mmol) and crude compound **5** in anhydrous methanol (10 mL) was added K_2CO_3 (0.91 g, 6.56 mmol) at 0 °C. The reaction mixture was stirred at 0 °C for 30 min, then warmed to room temperature and stirred for an additional 3 h. 10 mL of saturated aqueous NH_4Cl and 20 mL hexanes were added, the organic layer separated, dried over anhydrous $MgSO_4$, and concentrated. The residue was purified by column chromatography (EtOAc/hexane = 1/20) to give compound **6** as a colorless oil (356.31 mg, 41% over 2 steps). ^1H NMR (500 MHz, CD_3CN) δ 4.59-4.49 (m, 1H), 4.02 (dd, 1H, *J* = 8.8, 5.8 Hz), 3.93 (dd, 1H, *J* = 8.8, 1.85 Hz), 2.54-2.50 (m, 1H), 1.55 (s, 3H), 1.46 (s, 9H), 1.44 (s, 3H); ^{13}C NMR (125 MHz, CD_3CN) δ 152.31, 94.85, 84.25, 80.75, 71.27, 69.41, 49.18, 28.52, 27.53, 26.42, 25.23, 24.31; LC-MS calcd. for $C_{12}H_{20}NO_3$: 226.1443, found: 226.1451 *m/z* $[M + H]^+$.

(S)-2-aminobut-3-yn-1-ol ***((S)-AlkEA)***

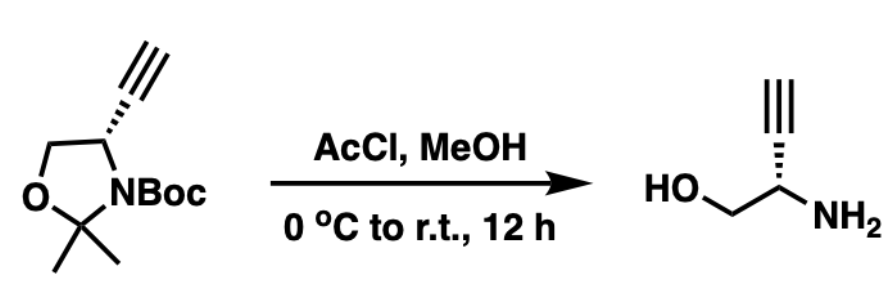

6 (*S*)-AlkEA

(***R***)**-AlkEA** was prepared according to literature procedures[4]. Briefly, to a solution of compound **3** (326.6 mg, 1.45 mmol) in dry methanol (2.2 mL) was added acetyl chloride (0.42 mL, 5.80 mmol) at 0 °C. The reaction was slowly warmed to room temperature and stirred for 12 h. The crude reaction was concentrated and purified by column chromatography (EtOAc/methanol = 7/3 plus 0.1M ammonia in ethanol) to give **(*S*)-AlkEA** as a yellow solid (117.1 mg, 95%). ^1H NMR (500 MHz, CD_3OD) δ 4.12 (ddd, 1H, *J* = 7.8, 4.2, 2.4 Hz), 3.87 (dd, 1H, *J* = 11.6, 4.2 Hz), 3.70 (dd, 1H, *J* = 11.6, 7.8 Hz), 3.22 (d, 1H, *J* = 2.4 Hz); ^{13}C NMR (125 MHz, CD_3OD) δ 78.39, 77.17, 63.08, 45.85; LC-MS calcd. for C_4H_8NO: 86.0606, found: 86.0619 *m/z* $[M + H]^+$.

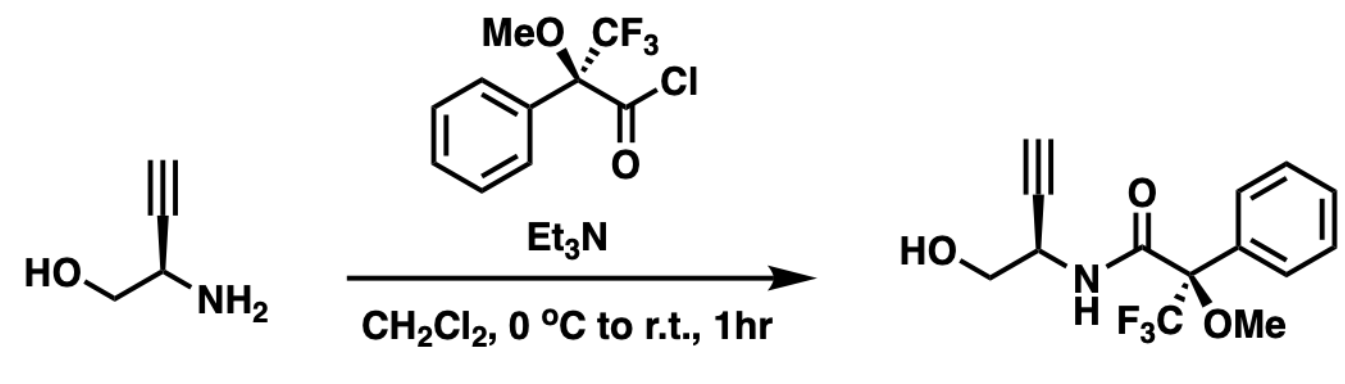

(R)-3,3,3-trifluoro-N-((R)-1-hydroxybut-3-yn-2-yl)-2-methoxy-2-phenylpropanamide (7) (for determination of ee of (R)-AlkEA)

To a solution of (*R*)-AlkEA (10.0 mg, 0.12 mmol) in dry CH_2Cl_2 (1.2 mL) was added Mosher's acyl chloride ((*S*)-(+)-α-methoxy-α-(trifluoromethyl)phenylacetyl chloride, 24.2 µL, 0.13 mmol) and triethylamine (25.1 µL, 0.18 mmol) at 0 °C. The reaction was slowly warmed to room temperature and stirred for 1 h. The crude reaction was concentrated and purified by column chromatography (hexane/EtOAc = 1/1) to give **7** as colorless liquid (19.9 mg, 55%). ^{1}H NMR (500 MHz, $CDCl_3$, both isomers) δ 7.53-7.47 (m, 2H), 7.42-7.37 (m, 3H), 7.30 (d, 1H, *J* = 7.7 Hz), 4.91-4.84 (m, 1H), 3.81-3.70 (m, 2H), 3.42-3.37 (m, 3H), 2.33 (d, 1H, *J* = 2.4 Hz), 2.30 (s, 1H); ^{13}C NMR (125 MHz, $CDCl_3$, both isomers) δ 166.17, 131.96, 129.64, 128.64, 127.71, 123.66 (q, *J* = 288.6 Hz), 84.00 (q, *J* = 26.3 Hz), 79.64, 73.01, 64.88, 54.94, 43.84; ^{19}F NMR (471 MHz, $CDCl_3$) δ -68.85 (minor isomer, 0.12 F), -68.96 (major isomer, 1.00 F), 79% ee; LC-MS calcd. for $C_{14}H_{13}F_3NO_3$: 300.0848, found: 300.0893 *m/z* $[M - H]^-$.

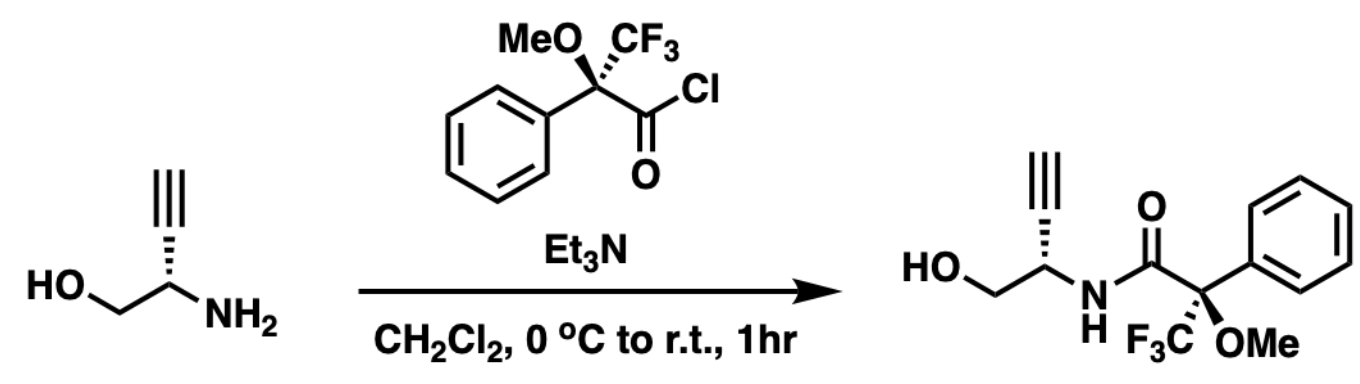

*(R)-3,3,3-trifluoro-N-((S)-1-hydroxybut-3-yn-2-yl)-2-methoxy-2-phenylpropanamide (**8**) (for determination of ee of (S)-AlkEA)*

To a solution of (*S*)-AlkEA (10.0 mg, 0.12 mmol) in dry CH_2Cl_2 (1.2 mL) was added Mosher's acyl chloride ((*S*)-(+)-α-methoxy-α-(trifluoromethyl)phenylacetyl chloride, 24.2 µL, 0.13 mmol) and triethylamine (25.1 µL, 0.18 mmol) at 0 °C. The reaction was slowly warmed to room temperature and stirred for 1 h. The crude reaction was concentrated and purified by column chromatography (hexane/EtOAc = 1/1) to give **8** as colorless liquid (18.06 mg, 50%). ^{1}H NMR (500 MHz, $CDCl_3$, both isomers): δ 7.53-7.48 (m, 2H), 7.41-7.36 (m, 3H), 7.23-7.20 (m, 1H), 4.91-4.83 (m, 1H), 3.79-3.66 (m, 2H), 3.43-3.37 (m, 3H), 2.35 (d, 1H, *J* = 2.4 Hz), 2.20 (s, 1H); ^{13}C NMR (125 MHz, $CDCl_3$, both isomers) δ 166.44, 132.21, 129.68, 128.69, 127.62, 123.67 (q, *J* = 288.5 Hz), 84.05 (q, *J* = 26.3 Hz), 79.52, 73.19, 64.92, 55.00, 43.88; ^{19}F NMR (471 MHz, $CDCl_3$) δ -68.85 (major isomer, 1.00 F), -68.95 (minor isomer, 0.13 F), 77% ee; LC-MS calcd. for $C_{14}H_{13}F_3NO_3$: 300.0848, found: 300.0855 *m/z* $[M - H]^-$.

NMR SPECTRA

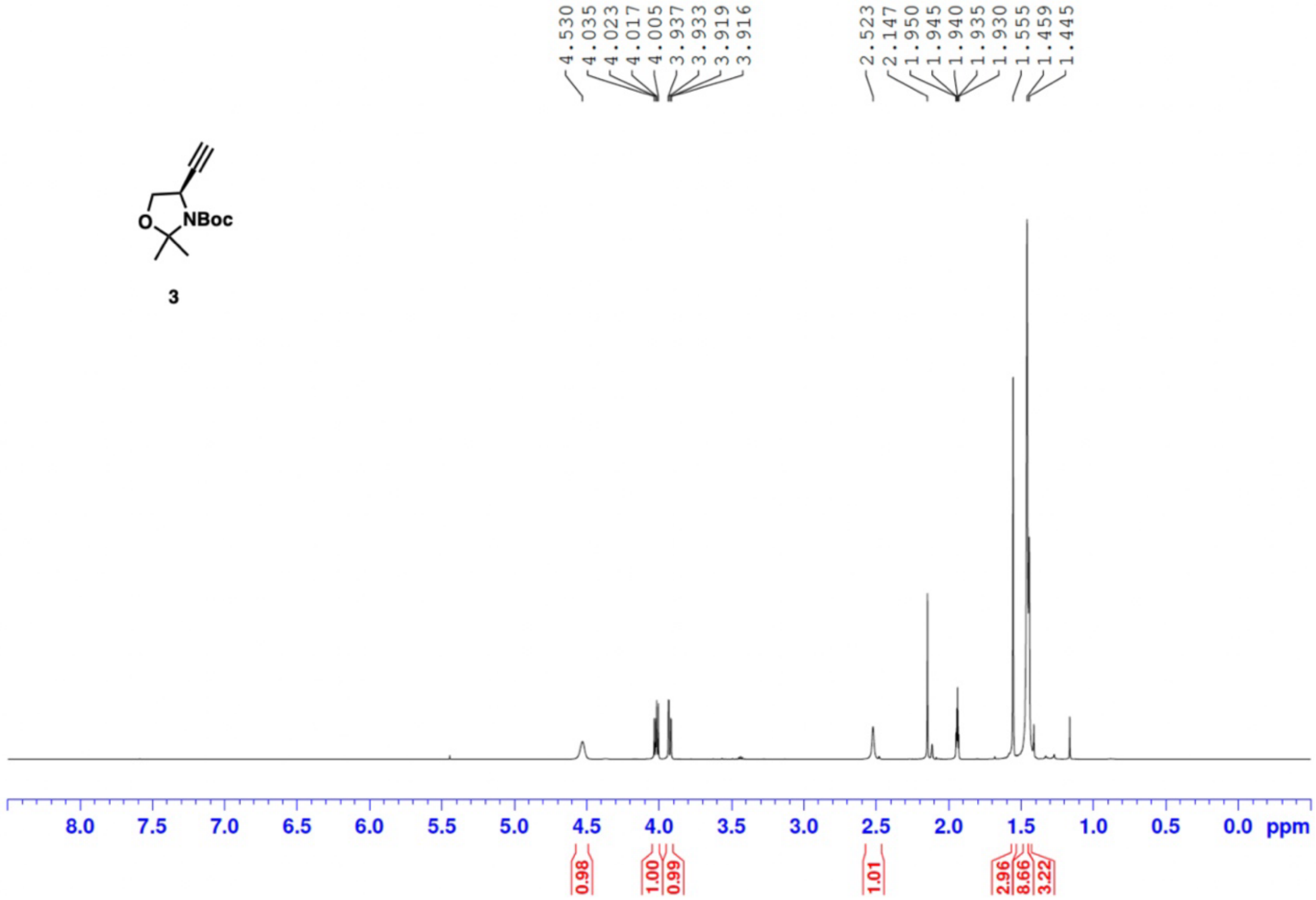

^{1}H NMR spectrum of compound **3**.

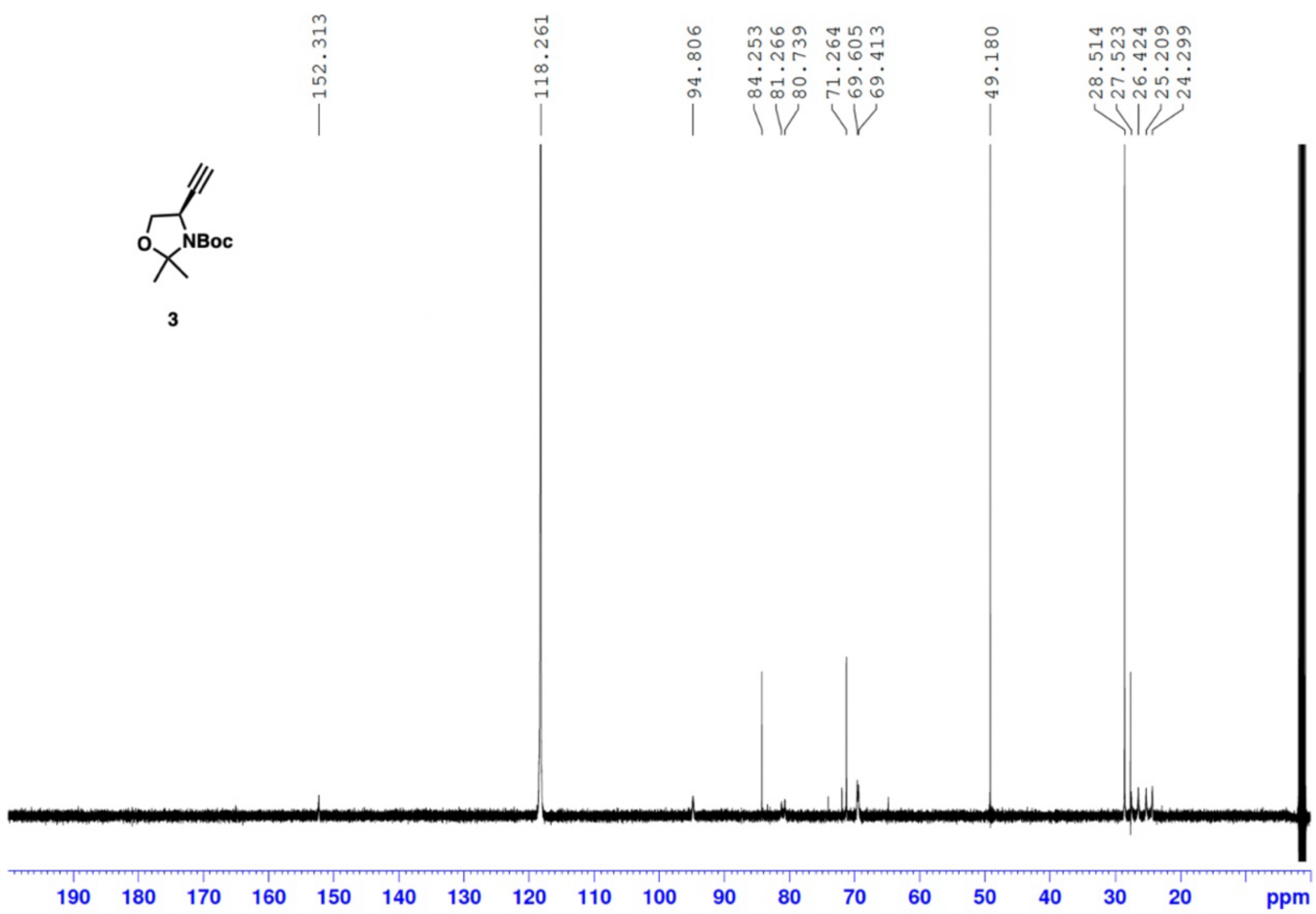

^{13}C NMR spectrum of compound **3**.

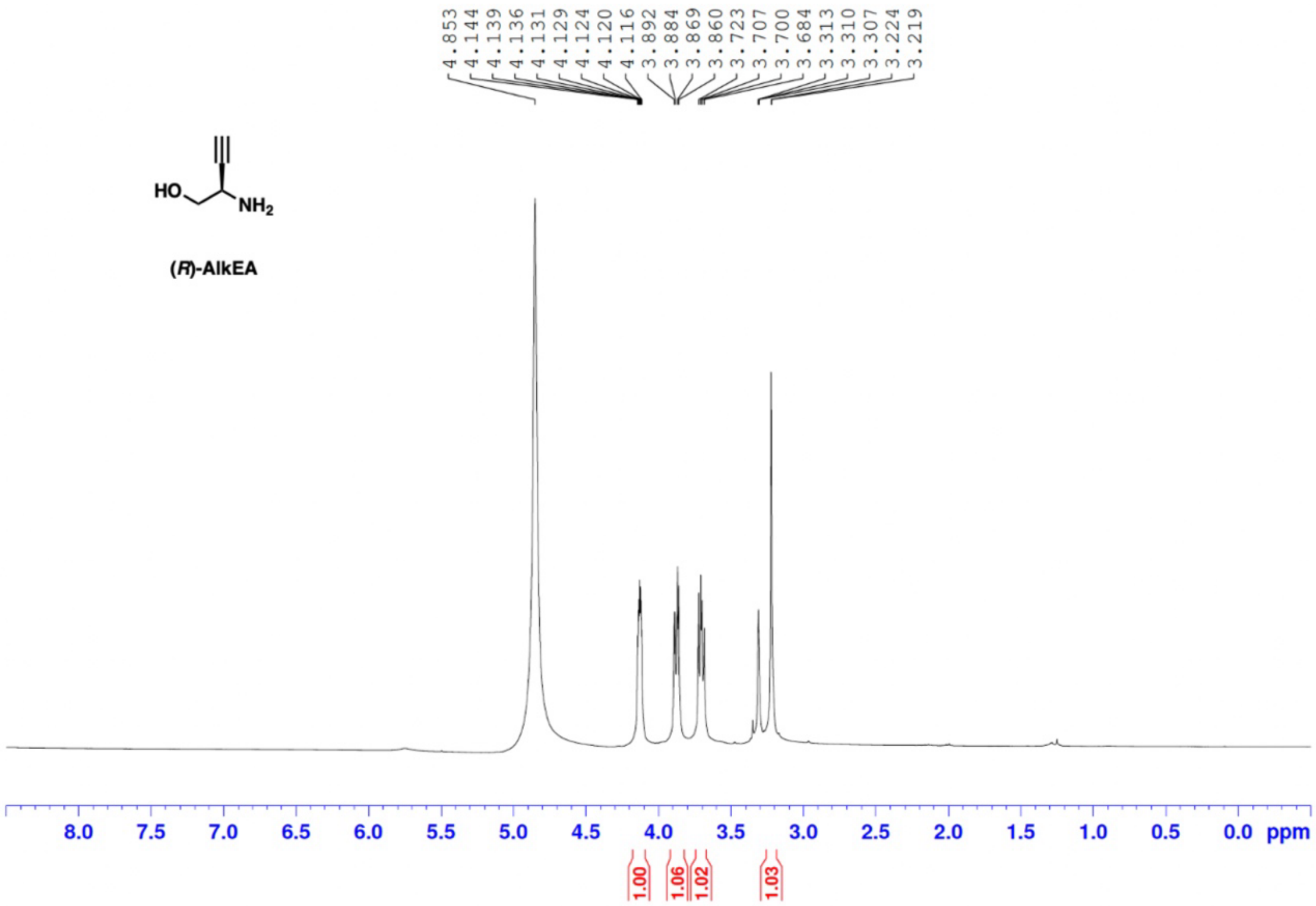

^{1}H NMR spectrum of ***(R)*-AlkEA**.

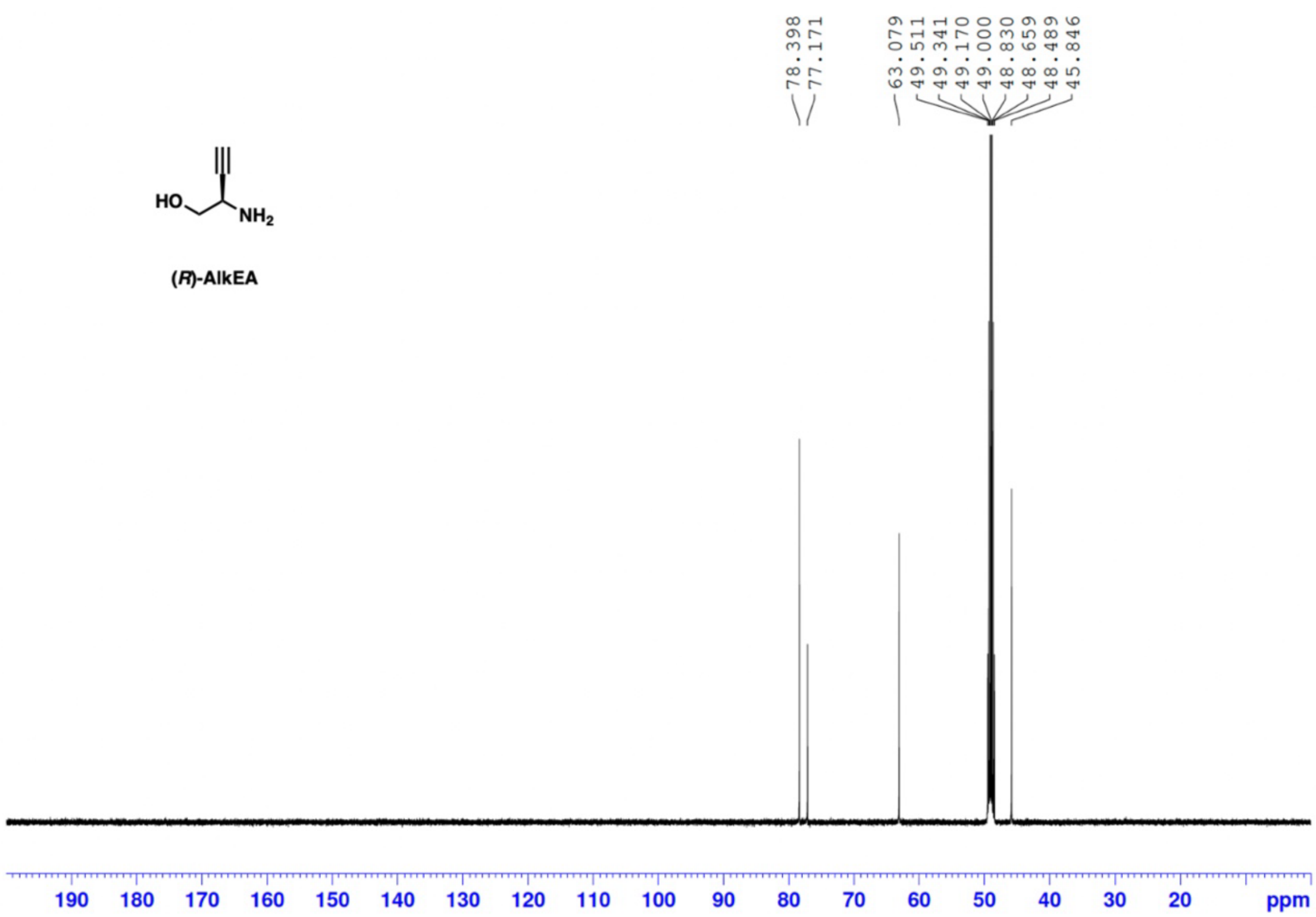

^{13}C NMR spectrum of ***(R)*-AlkEA**.

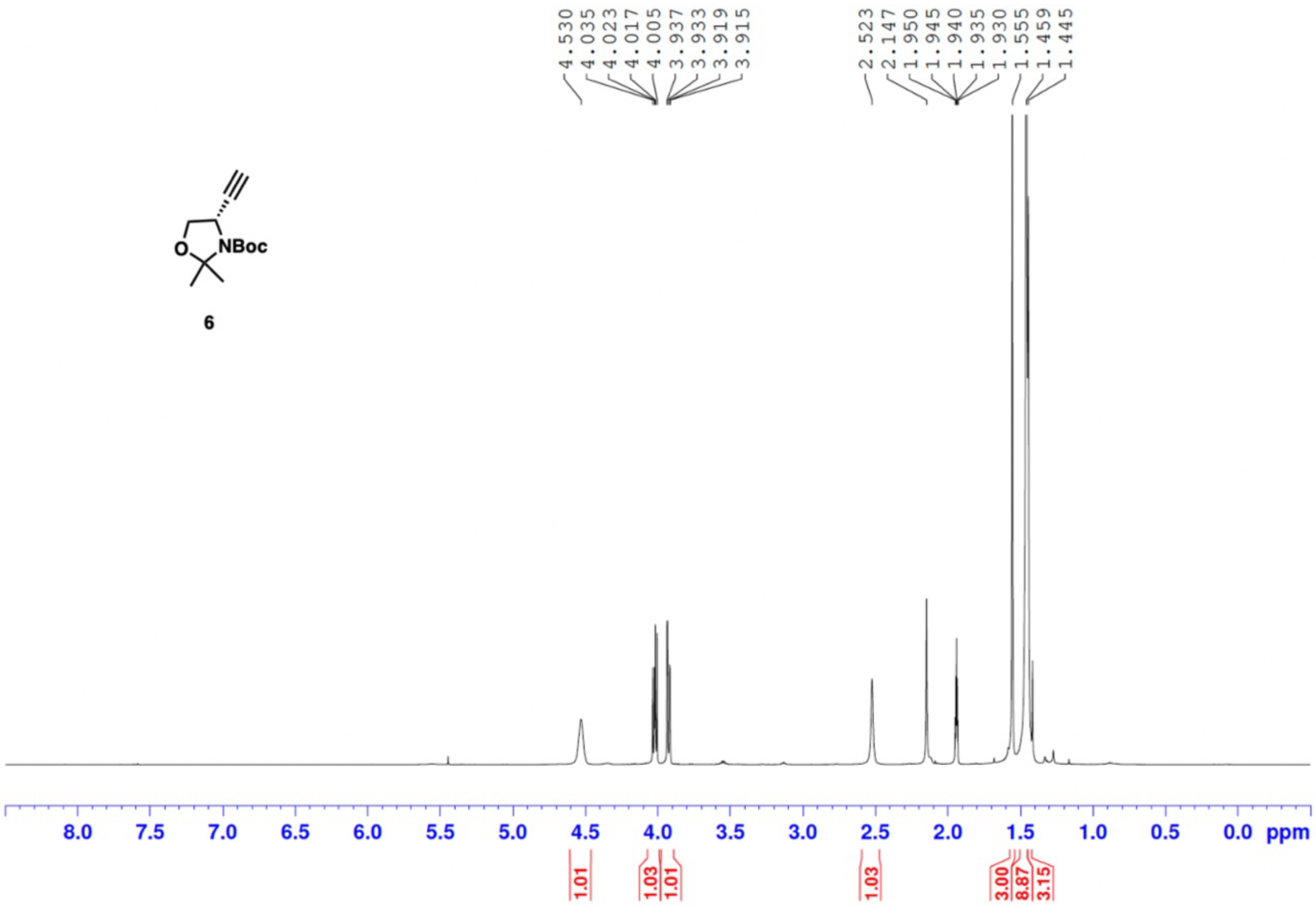

^{1}H NMR spectrum of compound **6**.

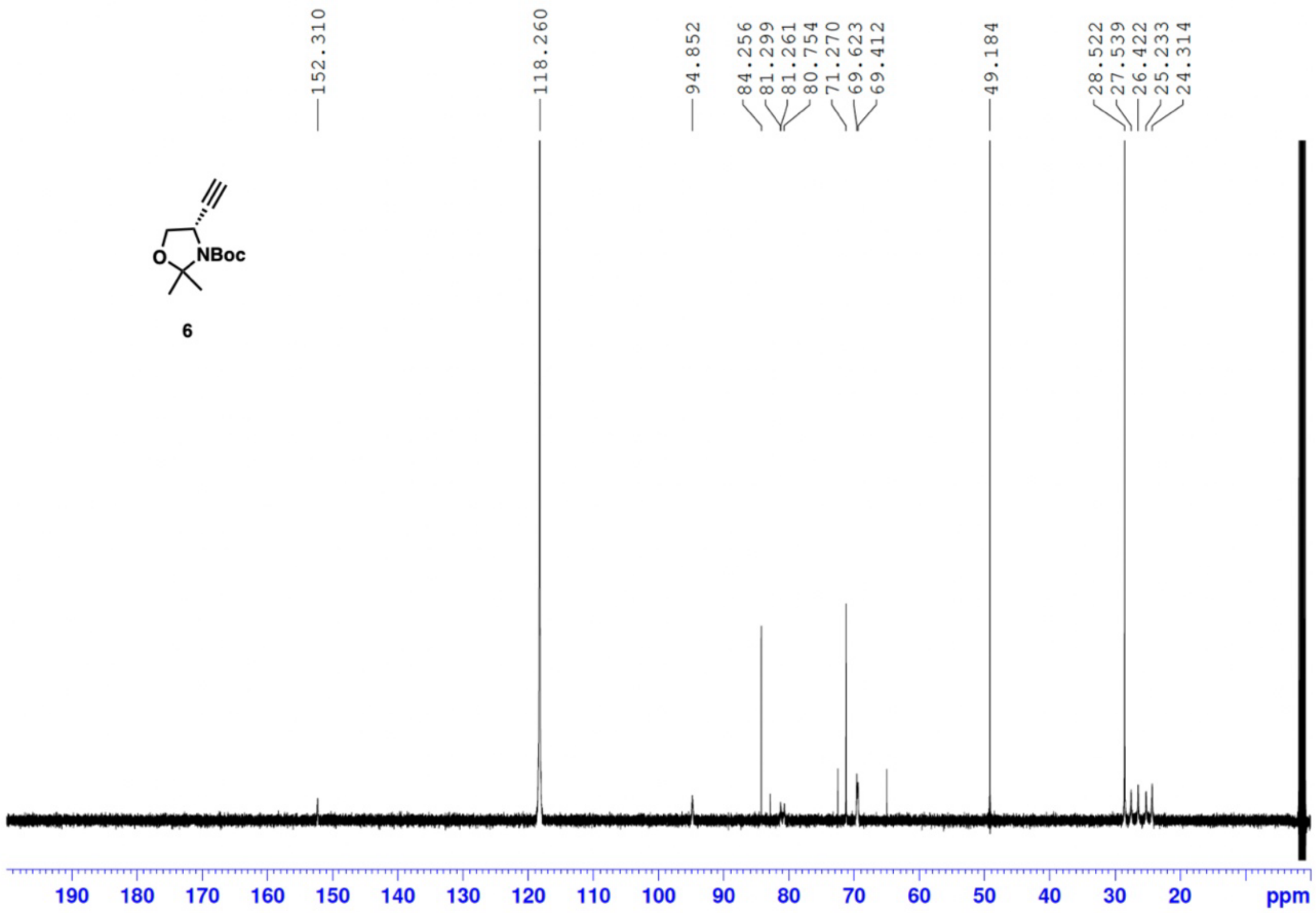

^{13}C NMR spectrum of compound **6**.

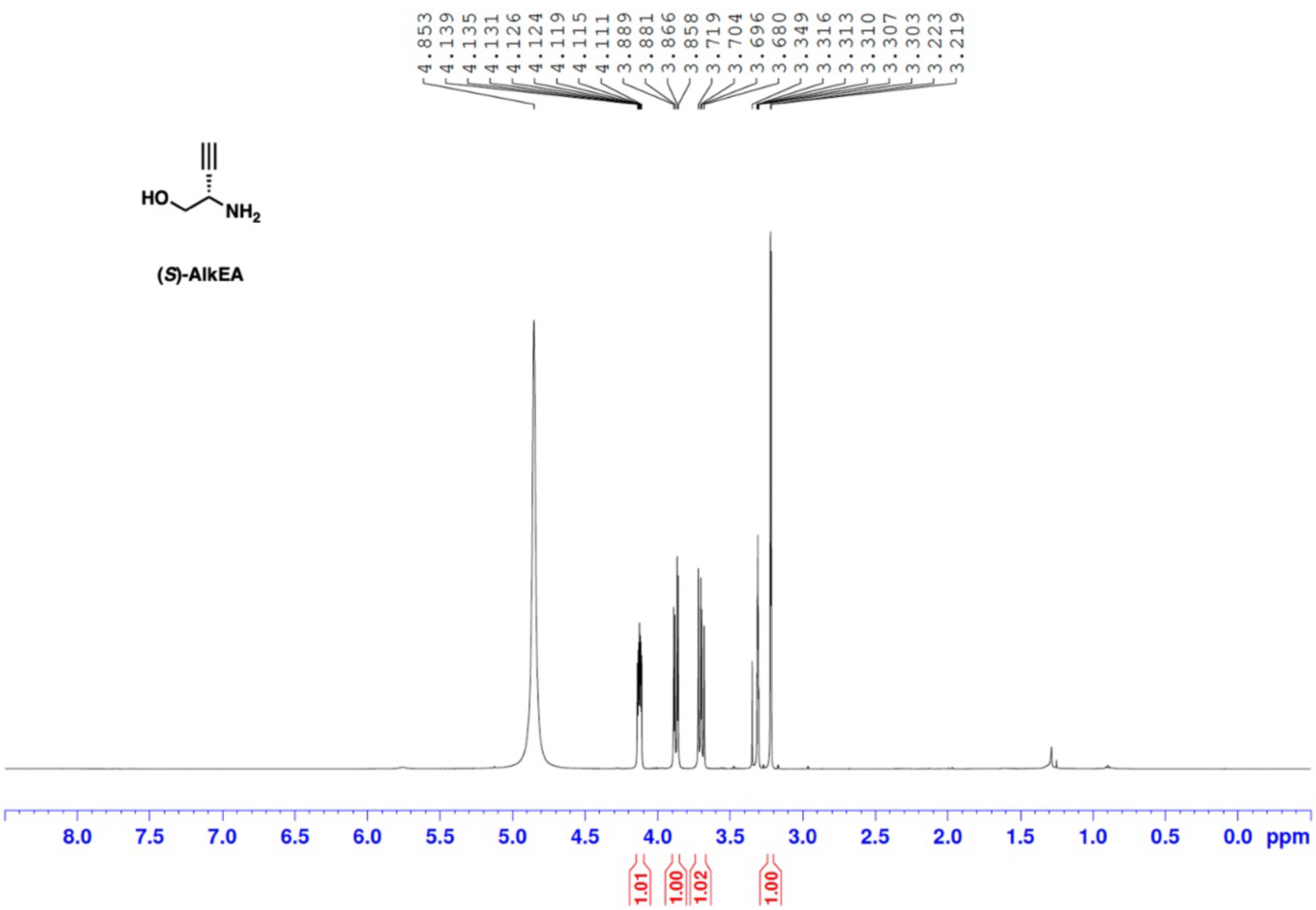

^{1}H NMR spectrum of ***(S)*-AlkEA**.

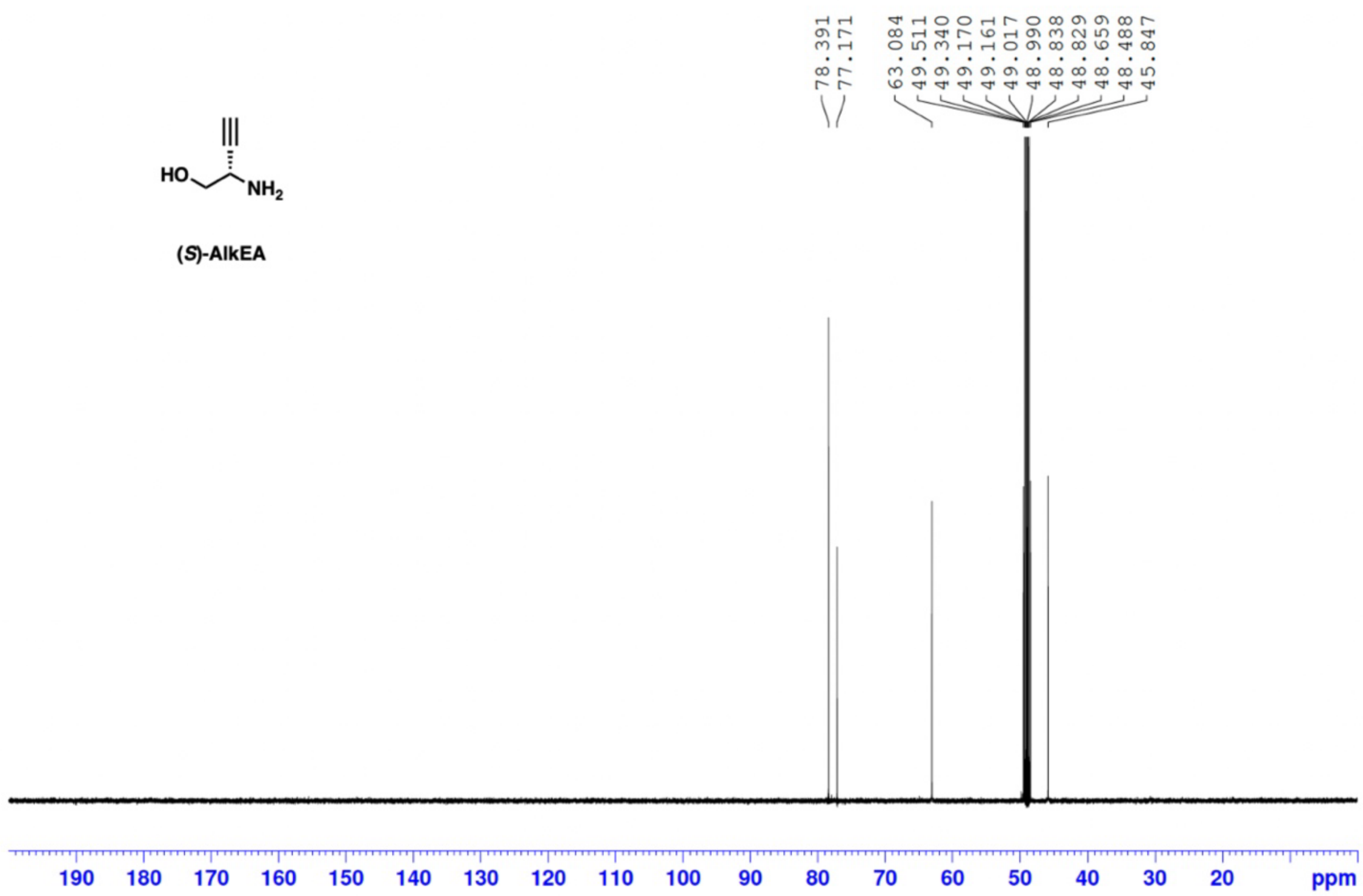

^{13}C NMR spectrum of ***(S)*-AlkEA**.

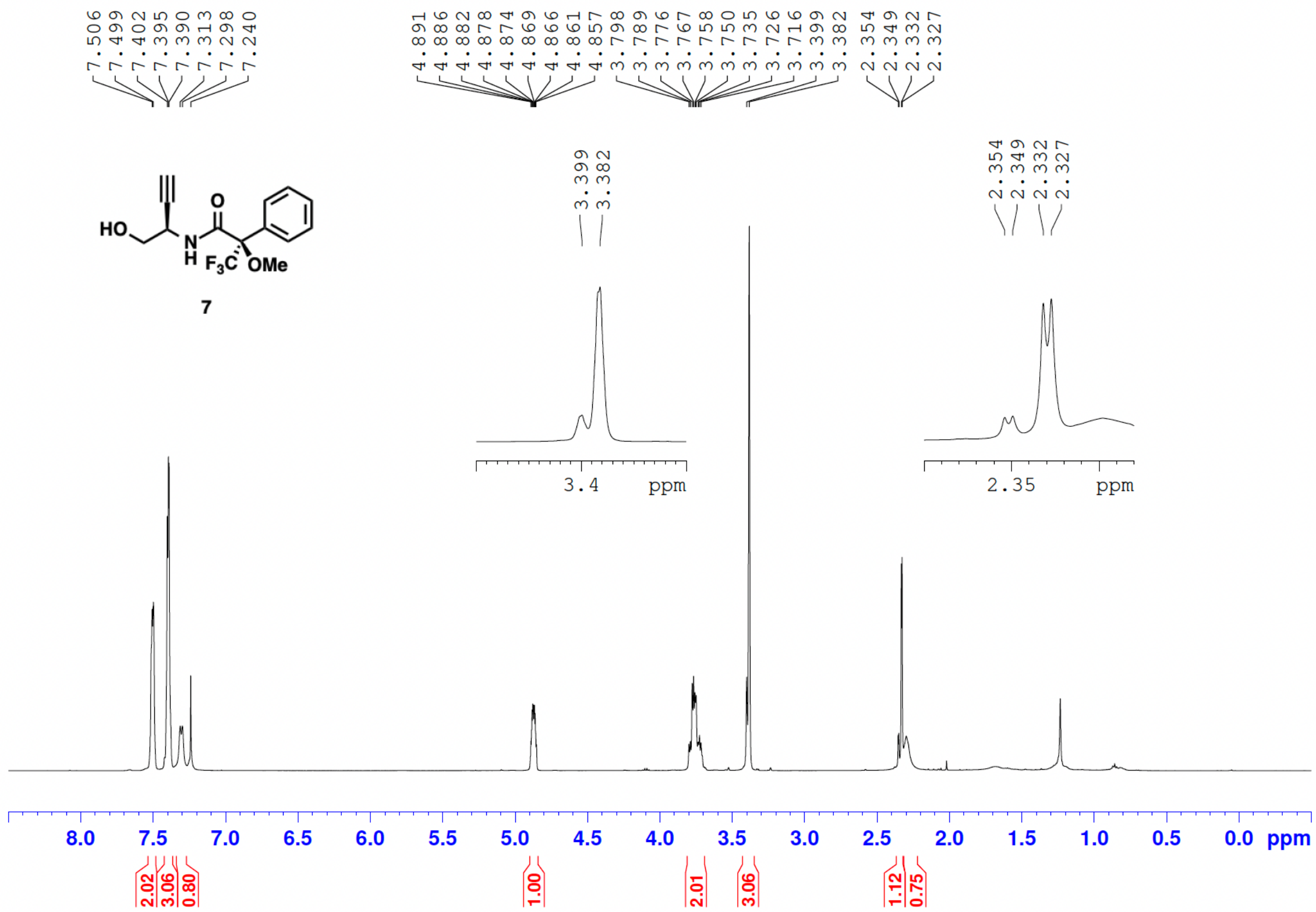

^{1}H NMR spectrum of compound **7**.

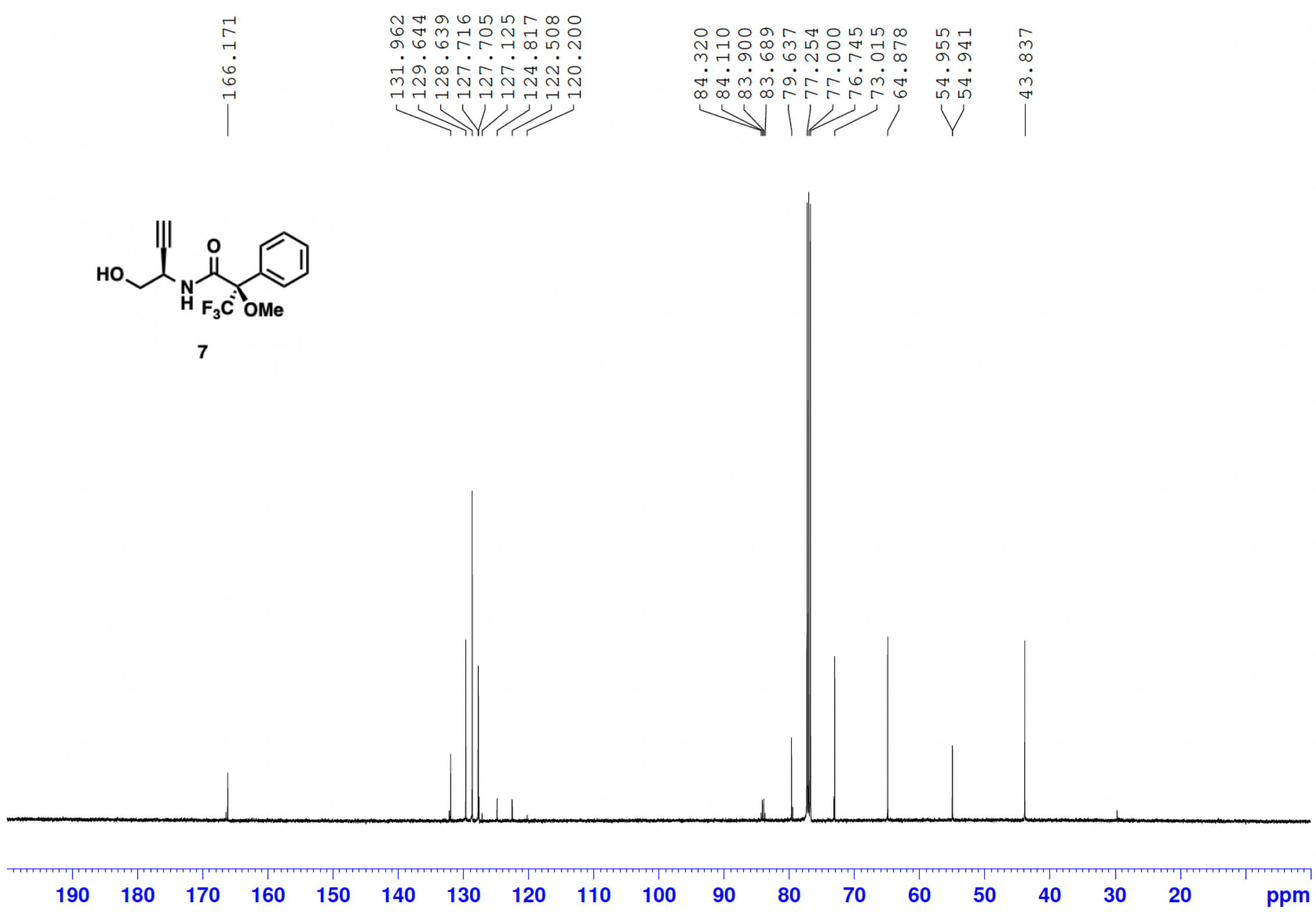

^{13}C NMR spectrum of compound **7**.

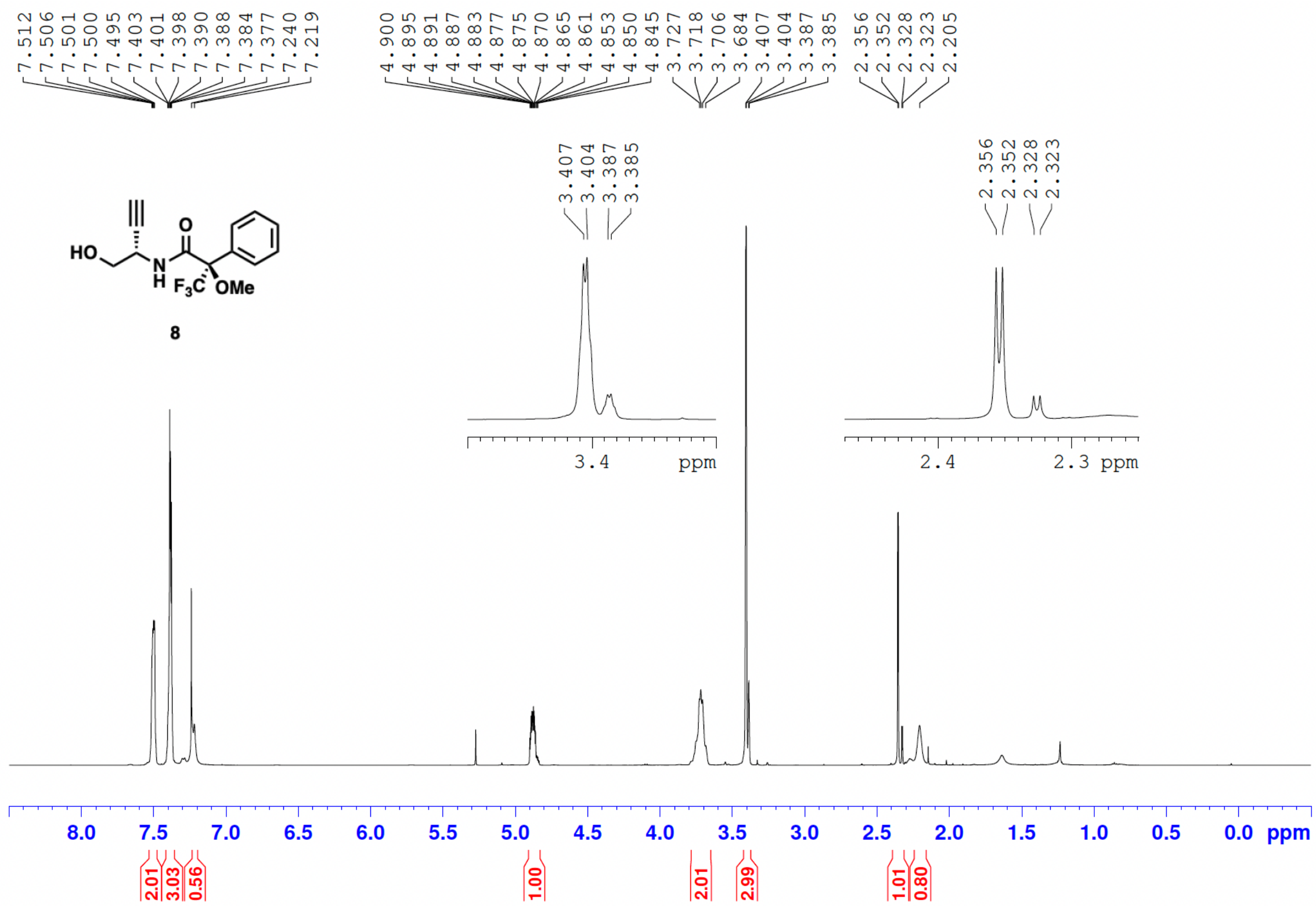

^{1}H NMR spectrum of compound **8**.

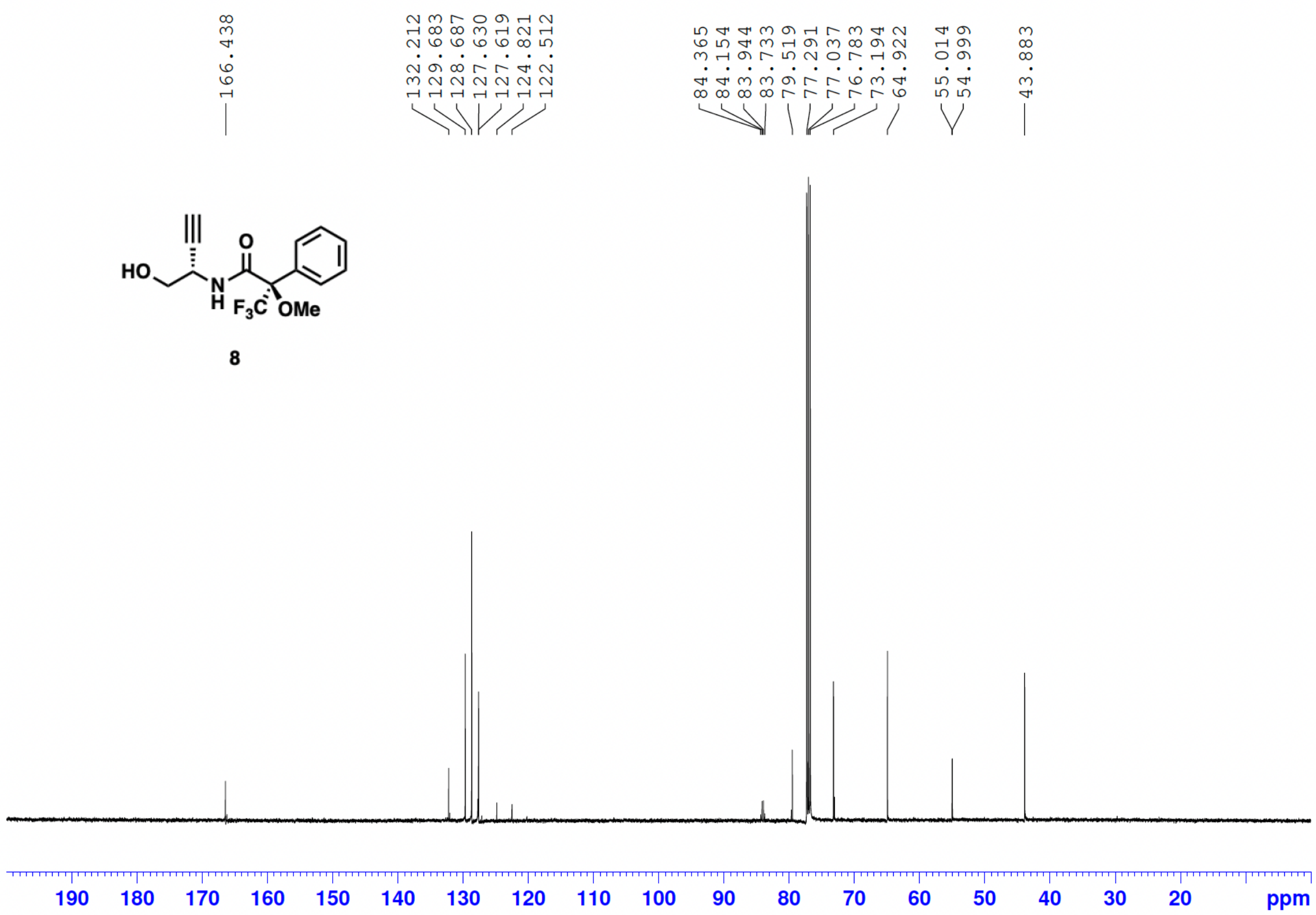

^{13}C NMR spectrum of compound **8**.

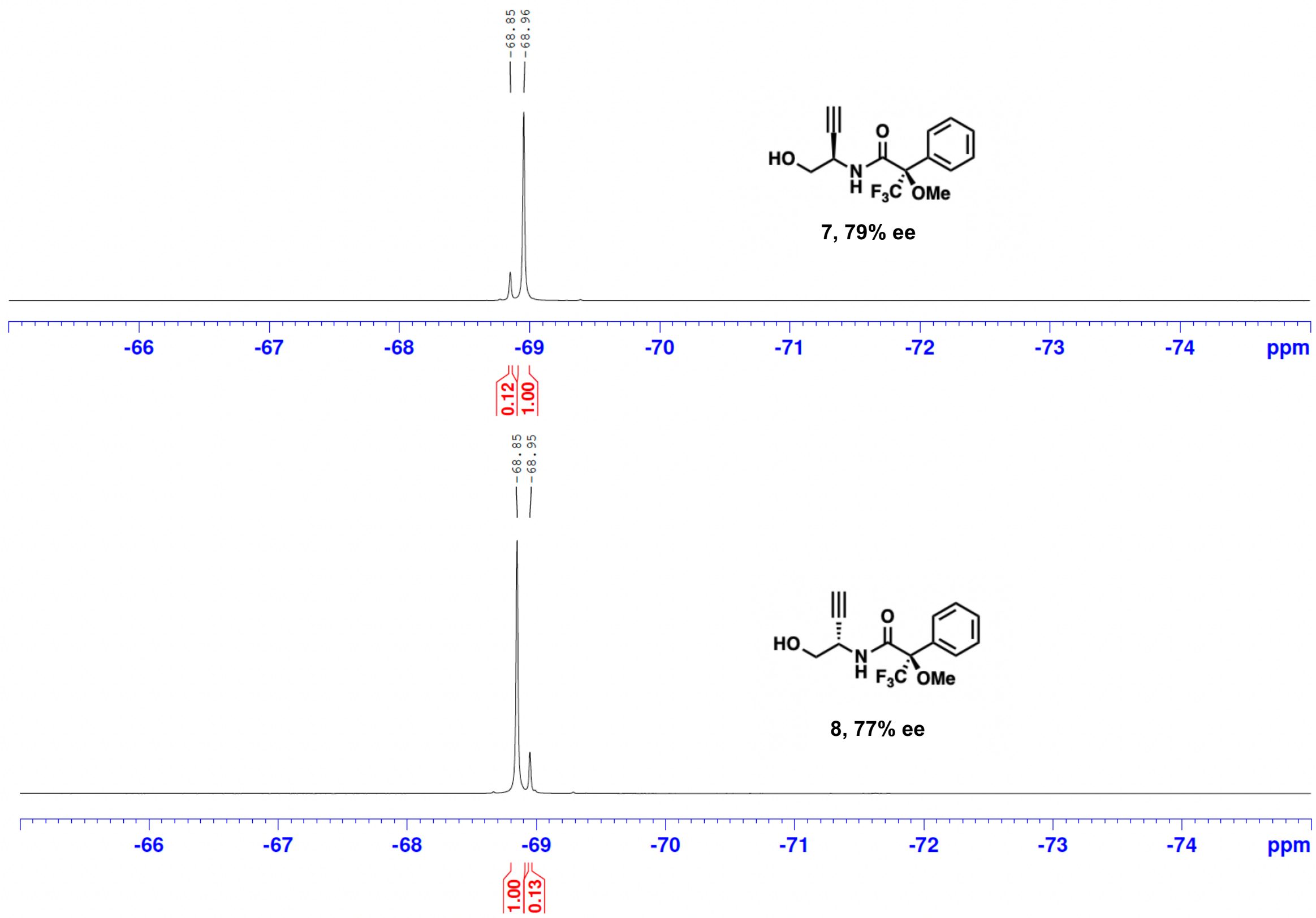

^{19}F NMR spectra of compound **7** and compound **8**.

REFERENCES

(1) Schindelin, J.; Arganda-Carreras, I.; Frise, E.; Kaynig, V.; Longair, M.; Pietzsch, T.; Preibisch, S.; Rueden, C.; Saalfeld, S.; Schmid, B.; Tinevez, J.-Y.; White, D. J.; Hartenstein, V.; Eliceiri, K.; Tomancak, P.; Cardona, A. Fiji: An Open-Source Platform for Biological-Image Analysis. *Nat. Methods* **2012**, *9* (7), 676–682. https://doi.org/10.1038/nmeth.2019.

(2) White, B. M.; Kumar, P.; Conwell, A. N.; Wu, K.; Baskin, J. M. Lipid Expansion Microscopy. *J. Am. Chem. Soc.* **2022**, *144* (40), 18212–18217. https://doi.org/10.1021/jacs.2c03743.

(3) Crisp, G. T.; Yu-Lin Jiang; Pullman, P. J.; De Savi, C. Elaboration of the Side-Chain of Amino Acid Derivatives by Palladium Catalysed Couplings. *Tetrahedron* **1997**, *53* (51), 17489–17500. https://doi.org/10.1016/S0040-4020(97)10197-1.

(4) Nudelman, A.; Bechor, Y.; Falb, E.; Fischer, B.; Wexler, B. A.; Nudelman, A. Acetyl Chloride-Methanol as a Convenient Reagent for: A) Quantitative Formation of Amine Hydrochlorides B) Carboxylate Ester Formation C) Mild Removal of N-t-Boc-Protective Group. *Synth. Commun.* **1998**, *28* (3), 471–474. https://doi.org/10.1080/00397919808005101.
